# Artificial intelligence driven diet‐intestinal microbiota‐host health integration: A four‐dimensional paradigm for advancing host wellness research

**DOI:** 10.1002/imo2.70133

**Published:** 2026-08-26

**Authors:** Tianle He, Shuobo Zhang, Jiaxin Chen, Xiaoling Zheng, Jixin Zhao, Huifeng Li, Jiani Mao, Ju Luo, Jundan Zheng, Dengjun Ma, Lihong Wang, Junhui Liu, Wen Tian, Shuangming Yang, Chengli Liu, Ke Tian, Zhenguo Yang

**Affiliations:** ^1^ College of Animal Science and Technology Southwest University Chongqing China; ^2^ Key Laboratory of Animal Nutrition and Bio‐feed Chongqing Municipal Education Commission Chongqing China; ^3^ College of Animal Science and Technology Ningxia University Yinchuan China; ^4^ Ningxia Key Laboratory of Precision Breeding and Nutritional Regulation of Characteristic Livestock and Poultry Ningxia University Yinchuan China; ^5^ Plant and Animal Genomics, Centre for Research in Agricultural Genomics (CRAG) CSIC‑IRTA‑UAB‑UB Consortium Bellaterra Spain; ^6^ Departament de Ciència Animal i dels Aliments, Facultat de Veterinària Universitat Autònoma de Barcelona (UAB) Bellaterra Spain; ^7^ Hunan Provincial Key Laboratory of Animal Nutritional Physiology and Metabolic Process, National Engineering Laboratory for Pollution Control and Waste Utilization in Livestock and Poultry Production, Institute of Subtropical Agriculture Chinese Academy of Sciences Changsha China; ^8^ College of Biology and Food Engineering Chongqing Sanxia University of Science and Technology Chongqing China; ^9^ The United Graduate School of Agricultural Science Gifu University Gifu Japan

**Keywords:** artificial intelligence, intestinal microbiota, host health, interaction‐mediated regulation, intestinal homeostasis

## Abstract

The artificial intelligence (AI)‐driven diet‐intestinal microbiota‐host health integration paradigm has emerged as a novel framework for advancing host health research. Intestinal microbiota serves as a key mediator linking dietary signals to host homeostasis, while AI enables efficient integration of multi‐omics data to construct microbial metabolic models, identify interaction patterns across biological scales, and support prediction‐informed modulation of diet‐microbiota interactions. This paradigm synergizes dietary intervention design, AI technology, intestinal microbiota modulation, and host health enhancement, offering innovative solutions for chronic disease management, animal health breeding, and food safety monitoring. In this review, we summarize the core mechanisms of the four‐dimensional interaction and the application value of AI‐driven informed optimization. This review also discusses the major challenges currently facing the field, including model interpretability, multi‐source data heterogeneity, and cross‐species translation, and further highlights that future research should focus on establishing interpretable and iterative AI‐driven closed‐loop systems. This integrated approach holds immense potential to revolutionize host wellness research and promote the development of nutritional health and related industries. Unlike existing diet‐microbiota‐host frameworks and AI‐assisted precision nutrition approaches that mainly focus on association analysis or outcome prediction, our proposed paradigm positions AI as an active coordination layer linking dietary inputs, microbial responses, and host outcomes. By integrating mechanism‐informed modeling with iterative feedback, this framework enables dynamic optimization of diet‐microbiota interactions and supports more precise host health regulation.

## Highlights

- Proposes an Artificial Intelligence (AI)‐driven four‐dimensional diet‐intestinal microbiota–host health paradigm.
- Elucidates AI advances in deciphering bidirectional diet–microbiota interactions.
- Evaluates AI‐driven precision nutrition intervention designs and clinical translation.
- Critically analyzes inherent limitations of microbiome AI models while outlining key challenges and future directions.

## Graphical abstract

This graphical abstract presents a novel Diet‐Artificial Intelligence (AI)‐intestinal Microbiota–Host Health paradigm integrating microbiota‐centric pathways and an AI‐driven multi‐omics workflow. Diet shapes gut bacteria that regulate intestinal mucosal integrity, immune signaling, and gut–brain neurotransmitters to influence host health. An AI multi‐omics pipeline processes multi‐omics data to construct knowledge graphs and design antimicrobial peptides, enabling predictive health modeling. This approach supports applications in personalized nutrition, food safety, and precision animal health. Key challenges include data silos, AI interpretability, and ethics, with future directions focusing on organoid models, spatial multi‐omics, digital twins, and multi‐modal deep learning to advance host wellness research.

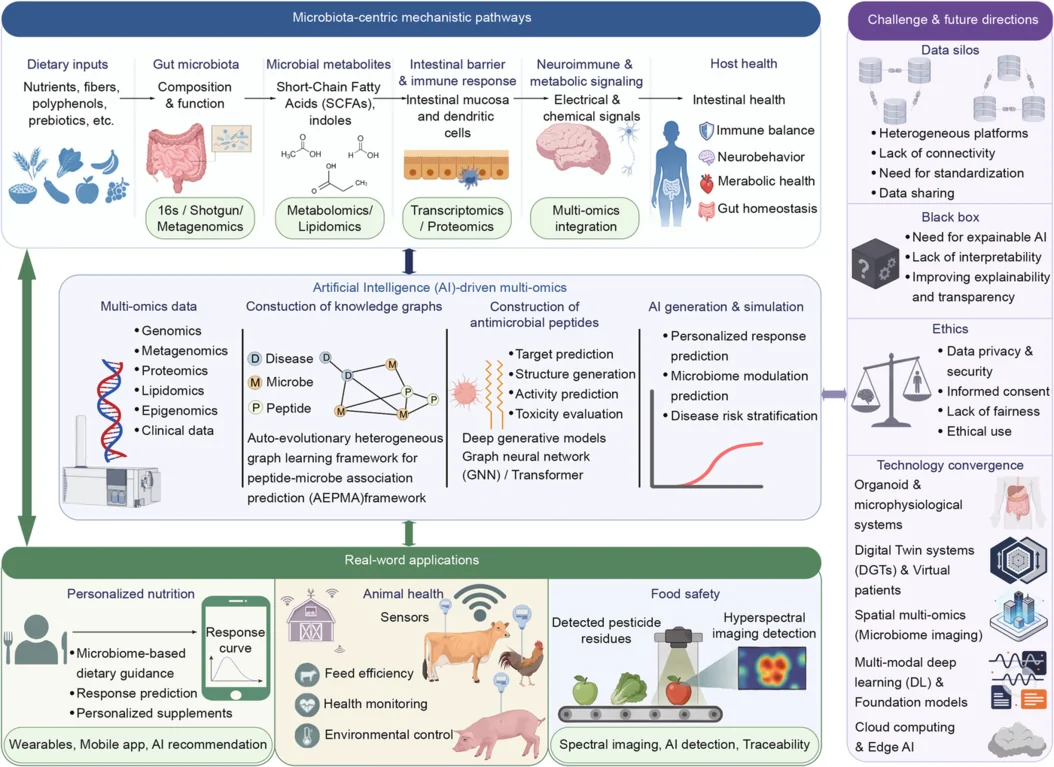

## 1 INTRODUCTION

The intestine, as the most microbially diverse ecological interface in the human body, establishes a tight symbiotic relationship with the host and plays a pivotal role in maintaining internal environment homeostasis, immune regulation, and metabolic balance [1]. As the most intervenable regulatory factor, diet serves as the core pathway for shaping the structure, function, and diversity of the intestinal microbiota. Breakthroughs in high‐throughput sequencing and integrated meta‐omics frameworks such as iMetaOmics have laid a solid data foundation for deciphering diet‐microbiota‐host health associations [2]. However, traditional analytical methods struggle to cope with such high‐dimensional and nonlinear complex relationships, and the rise of artificial intelligence (AI) has provided a transformative tool. With the rapid advancement of deep learning (DL), large language models (LLMs), multimodal fusion, and knowledge graph reasoning, AI not only accelerates large‐scale data processing but also supports the generation of biologically informed hypotheses through inference models, thus emerging as an independent new research paradigm [3]. AI refers to computational systems that emulate aspects of human cognition and decision‐making through algorithmic and data‐driven processes [4]. Among its major branches, ML enables computers to identify latent patterns from complex datasets and continuously improve predictive performance through iterative learning. Given the diversity of AI methodologies and their distinct analytical capabilities, selecting appropriate models according to specific biological questions and application scenarios is essential [4, 5, 6, 7, 8, 9, 10, 11, 12, 13, 14, 15, 16, 17, 18, 19, 20, 21, 22, 23, 24, 25, 26, 27, 28, 29, 30, 31, 32, 33, 34, 35, 36, 37, 38, 39, 40, 41, 42, 43, 44, 45, 46, 47, 48, 49, 50] (Table 1). Importantly, AI‐derived outputs are predominantly based on statistical associations rather than direct causal inference and therefore require further biological interpretation and experimental validation. AI has been widely applied in scenarios including microbial phenotype prediction, probiotic characteristic analysis, nutritional phenotype modeling, and functional dietary component screening. In the field of microbial research, the application of machine learning (ML) and DL is becoming increasingly prevalent. CnnPOGTP enables accurate prediction of the optimal growth temperature of microorganisms; CheckM2 assesses the quality of MAGs; iProbiotics and metaProbiotics are used for probiotic trait prediction; and image‐based AI recognition technology has significantly improved the efficiency of automated screening of microbial colonies [51]. In terms of nutrition and health, Ramyaa et al. [52] employed ML to analyze nutritional phenotypes, successfully predicting body weight and body mass index in female participants based on dietary components and physical activity variables. The model developed by Berry et al. [53] can predict individual postprandial serum triglyceride and glucose responses. ML‐based computational prediction has also made it feasible to identify dietary components with immunomodulatory, anti‐aging, and other potentials, while clarifying their underlying mechanisms. Rein et al. [54] screened immunomodulatory peptides from rice proteins through this approach and verified their bioactivity. Additionally, studies have reported ML models for analyzing changes in intestinal microbiota and metabolites following intervention design with dietary components such as prebiotics [5, 55]. These interdisciplinary studies are driving the transformation of the diet‐microbiota‐host health network from observational research to systematic modeling.

**Table 1:** Types of artificial intelligence (AI).

AI method	Biological question	References
Random Forest (RF)	Disease diagnosis, nutritional assessment, gut microbial colonization prediction	[4, 5, 6, 7, 8, 9]
Support Vector Machine (SVM)	Food classification, pesticide residue detection, meat adulteration, and disease auxiliary diagnosis	[10]
Artificial Neural Networks (ANNs)	Real‐time monitoring in food processing, Antimicrobial Peptides (AMPs) activity prediction, functional annotation of microbial proteins	[15, 16, 17, 18]
Convolutional Neural Networks (CNNs)	Food image grading, contaminant monitoring, and foodborne pathogen identification	[19, 20, 21, 22, 23]
k‐Nearest Neighbors (KNNs)	Food safety detection, quality control	[24]
Gradient boosting models	Gut metabolite prediction, food spoilage risk, hepatocellular carcinoma differentiation	[25, 26, 27]
Long Short‑Term Memory (LSTM)	Novel AMPs identification, spatiotemporal dynamics analysis of microbial communities, time‐series prediction of processing parameters	[28, 29]
Machine Learning (ML)	Disease diagnosis, microbial community clustering, personalized nutrition dynamic optimization	[4, 18, 30, 31]
Natural Language Processing (NLP)	Identification of associations between emotional eating behavior and mental health	[32, 33]
Text and speech synthesis	Voice control and command feedback for equipment in farming scenarios	[34]
Computer Vision (CV)	Animal behavior and health monitoring, food adulteration detection	[35, 36, 37, 38, 39, 40]
Robotics	Assistance in minimally invasive surgery, patient rehabilitation, automatic feeding, and food sorting	[41]
Planning and expert Systems (ES)	Risk warning in food supply chains, formulation of personalized nutritional intervention design plans	[42, 43, 44, 45]
Graph Neural Networks (GNNs)	Microbial interaction networks, gut co‑metabolic networks, supply chain risk analysis	[42, 46, 47]
Generative Adversarial Networks (GANs)	De novo design of AMPs, food image data augmentation, and optimized design of packaging	[48, 49, 50]

From a One Health perspective, the diet‐AI‐microbiota‐host framework extends beyond single‐host mechanisms to human, animal and environmental health systems [56]. As downstream application scenarios of this framework, the “One Health” concept, cross‐species health, and agricultural and food systems represent important areas in which AI‐driven paradigm innovation is reshaping current research and application strategies. These advances not only provide technical support for understanding the interconnections between human and animal health but also enable the transfer and validation of diet‐microbiota‐host interaction mechanisms across different biological systems. The “One Health” concept further expands this framework from an ecosystem‐level perspective by integrating human, animal, and environmental health into a unified context. Against the backdrop of escalating global health security challenges, increasing pressure on food systems, and the growing threat of antimicrobial resistance, such cross‐species and cross‐scale health interactions have become particularly important. In practical applications, the ability of AI to identify and generate testable hypotheses regarding diet‐microbiota‐host interactions may be further extended to fields such as animal nutrition, disease prevention and control, and food safety. In agricultural production, the AI‐driven DSAP framework (De Novo Domestication‐Smart Breeding‐AI Phenomics) [57] accelerates the entire crop improvement pipeline by integrating germplasm innovation, breeding acceleration, and phenotypic evaluation, thereby promoting sustainable agricultural development while ensuring nutritional supply. Collectively, these findings suggest that AI not only contributes to host health regulation, but also participates in the regulation of food production systems, thereby promoting the large‐scale optimization of the diet‐microbiota‐host network across the food‐health continuum.

In this review, we conceptualize this framework as comprising three biological dimensions‐diet, intestinal microbiota, and host health integrated through a cross‐cutting AI computational dimension. Diet represents exogenous nutritional inputs and bioactive compounds that initiate host–microbiota interactions. Intestinal microbiota functions as the central biological mediator linking dietary signals to physiological responses, whereas host health reflects the resulting metabolic, immunological, and neurophysiological outcomes. Distinct from these biologically defined dimensions, AI is introduced as a computational integration and coordination layer that enables multi‐source data fusion, hypothesis generation, predictive modeling, and iterative optimization across the diet–microbiota–host axis. Therefore, rather than representing four equivalent biological dimensions, the proposed framework comprises three biological dimensions (diet, intestinal microbiota, and host health) integrated by AI as a cross‐cutting computational dimension.

Building on the aforementioned context, this review aims to establish an AI‐driven four‐dimensional integrated paradigm centered on the “diet‐intestinal microbiota‐host health” axis. Throughout this review, AI is regarded as a cross‐cutting computational dimension rather than an independent biological component. It functions as the analytical layer that integrates, models, and optimizes interactions among diet, intestinal microbiota, and host health. Taking intestinal microbiota metabolites as the core bridge linking nutrition and health, and integrating the regulatory mechanisms of gut‐X axis signaling networks, where “X” refers to distal organs such as the brain, liver, lung, and heart, serves as the core analytical framework throughout the article. Within this framework, the review first elucidates the pivotal role of gut microbial metabolites in linking dietary inputs to host health outcomes and further examines the regulatory mechanisms underlying gut‐axis signaling networks. On this basis, it systematically summarizes AI‐based technological systems, including multi‐omics integration, knowledge graph construction, and generative design, with particular emphasis on their capabilities in association identification and hypothesis generation within complex biological systems. Furthermore, the framework is extended to representative application scenarios such as personalized nutrition, healthy breeding, and food safety, thereby illustrating its transferability and practical potential across different biological and industrial systems. At the same time, this review discusses the major technical bottlenecks and ethical governance challenges associated with current AI applications, and proposes future‐oriented pathways for interdisciplinary integration and development. Importantly, rather than treating these limitations as an isolated future challenge, discussions of model interpretability, sample constraints, data heterogeneity, and causal limitations are integrated throughout each application scenario to provide a balanced evaluation of translational readiness. Ultimately, this work provides theoretical support for constructing precise AI‐enabled dietary microbiota regulatory strategies for host health [58]. The novelty of this framework lies not in introducing an additional dimension, but in redefining AI from an analytical tool into a mechanistic and iterative coordination layer across diet–microbiota–host interactions.

## 2 MICROBIOTA MEDIATES NUTRITIONAL SIGNALING AND LINKS TO HOST HEALTH

### Short‐chain fatty acids (SCFAs) and other intestinal microbiota metabolites mediate host energy and neural regulation processes

SCFAs and microbiota‐biotransformed dietary phytochemicals are key mediators linking dietary components to host metabolism, immunity and neural function [59]. However, conventional analytical approaches often struggle to identify the microbial taxa responsible for SCFA production and to resolve their complex interactions with host physiology. AI has therefore emerged as a powerful tool for integrating complex microbiome datasets, identifying functional microbial signatures, and generating mechanistic hypotheses. Emerging studies have demonstrated that AI‐assisted analytical frameworks can effectively address these challenges. For example, in studies investigating SCFA‐associated intestinal health mechanisms, AI has been applied to identify key microbial taxa involved in SCFA biosynthesis from large‐scale microbiome datasets. A study by Yin et al. [60] employed machine learning models, including gradient boosting, to analyze globally collected intestinal MAG datasets, identifying microbiome features associated with Enterobacteriaceae colonization from a vast number of microbial species and genes. This approach efficiently screened microbial taxa potentially involved in SCFA biosynthesis and revealed that SCFAs‐producing bacteria, such as *Faecalibacterium*, exhibited co‐exclusionary relationships with Enterobacteriaceae, suggesting a potential role in maintaining intestinal ecological stability. These findings indicate that AI is evolving beyond conventional statistical analysis toward the identification and inference of functional associations. Nevertheless, identifying candidate microbial taxa at the community level alone remains insufficient to fully elucidate the specific mechanisms through which SCFAs regulate host energy metabolism. Following the identification of key microbial populations, integrated multi‐omics analyses combined with experimental validation are still required to further investigate their potential downstream regulatory pathways. Similarly, Guo et al. [61] collected fecal samples from patients with myalgic encephalomyelitis/chronic fatigue syndrome (ME/CFS) and healthy controls. Using DNA sequencing technology and quantitative polymerase chain reaction (qPCR), they found that the patients exhibited insufficient capacity of intestinal microbiota to synthesize butyric acid, while the underlying mechanisms remained elusive. In another subsequent AI‐integrated study focusing on ME/CFS [62], BioMap AI was applied to integrate 4 years of multi‐omics data, including intestinal MAGs and plasma metabolomics, to construct a multi‐omics interaction map comparing healthy individuals and ME/CFS patients. When combined with immune cell data, this study clarified that SCFAs may participate in the inflammatory response processes and mechanisms of ME/CFS by regulating the secretion of inflammatory factors in immune cells. Beyond SCFAs, AI can also screen a broader range of microbial metabolic active molecules, facilitating the discovery of relevant targeted intervention design strategies from natural products (NPs). The conventional AHP‐SOM algorithm [63] can be used to screen the core active components of Jiedu Sangen Decoction (JSD) against colorectal cancer (CRC), identifying three core components, such as resveratrol and their corresponding targets. Experimental validation confirmed that JSD exerted a strong pro‐apoptotic effect on CRC in vitro, establishing a closed‐loop framework from algorithmic prediction to biological verification. However, these approaches remain largely constrained by predefined evaluation criteria and expert‐driven feature selection. Beyond this conventional framework, AI‐driven methodologies provide a novel strategy by enabling data‐driven identification of latent interaction patterns within diet–microbiota–host systems. Rather than simply prioritizing candidate compounds, AI models are capable of integrating heterogeneous datasets to infer compound–microbiota–host interaction networks, thereby uncovering functional relationships and potential mechanistic associations that may not be explicitly represented in existing knowledge systems.

ML approaches can integrate chemical characteristics with microbial genomic information to quantitatively predict the strength of compound–microbe interactions, thereby moving beyond conventional candidate prioritization strategies [64]. Meanwhile, AI‐based frameworks are capable of modeling host‐microbiome systems as complex interaction networks, enabling the identification of previously unrecognized associations between microbial and host components [65]. To improve the interpretability of microbiome AI models, Dawkins and Gerber developed MMETHANE, an interpretable deep learning framework that jointly analyzes microbial composition and metabolomics data while generating human‐readable rules linking microbes, metabolites, and host status [66]. In addition, deep learning models can generate latent representations linking microbiome and metabolome datasets, allowing the inference of microbe–metabolite interaction architectures rather than relying solely on predefined associations [67]. Collectively, these advances reflect an important transition from traditional feature‐based prioritization toward data‐driven inference of complex biological interaction networks.

The impacts of microbial metabolites on the host extend further to the levels of epigenetics and the central nervous system. Propionic acid and butyric acid can be converted into acyl‐CoA, which catalyzes the formation of non‐canonical histone modifications such as H3K18pr and H4K12bu, thereby increasing chromatin accessibility and regulating gene expression. Specifically, propionic acid accumulates in the regions of genes associated with cell migration, while butyric acid inhibits proliferation‐related genes in CRC cells and enhances the expression of barrier function‐related genes in normal intestinal epithelial cells [68]. Metabolomic experiments designed to directly measure these impacts are not only challenging but also costly; in contrast, computational methods that predict metabolomic profiles from microbial composition can significantly save experimental efforts. Consequently, these microbiota‐host interaction mechanisms also underpin microbiota‐targeted longevity interventions, which are increasingly supported by AI‐driven models [69]. Wang et al. [70] developed a method named Metabolomic Profile Predictor based on deep neural network architectures using modified Neural Ordinary Differential Equations (mNODE). This approach is capable of predicting metabolomic characteristics of both human and environmental microbiomes while incorporating dietary information to identify and generate testable hypotheses regarding microbe–metabolite interactions. The model provides a data‐driven framework for simulating the dynamic changes of metabolites such as SCFAs and their associations with specific microbial taxa. However, these findings are still primarily derived from association‐based modeling and therefore require further experimental validation to confirm their biological significance. On the other hand, Wu et al. [71] employed the Random Forest (RF) model to construct Twin Competitive Guilds (TCGs) composed of beneficial microbial taxa and potentially harmful microbial taxa based on the abundance profiles of high‐quality MAGs (HQMAGs). This strategy transformed complex microbial signals into more interpretable outcomes and provided a basis for the development of potential intervention design strategies, thereby improving the translational potential of AI‐derived predictive results to practical applications. Furthermore, intestinal microbes and their metabolites can also participate in energy metabolism [72] and immune homeostasis regulation [73] by regulating the expression of host long non‐coding RNAs (LncRNAs) and adipocyte thermogenic pathways [74], while changes in dietary structure can participate in host developmental processes by influencing the expression of circular RNAs (circRNAs) [75], providing a new connection pathway for microbial effects on phenotypes.

In summary, AI can be applied across multiple components of the nutritional metabolic transformation axis, including microbial bioactive metabolites such as SCFAs, epigenetic regulation, and the central nervous system. These models operate at different analytical levels, ranging from molecular to community and system scales, each with distinct strengths and the potential for functional complementarity. If systematically developed and experimentally evaluated in future studies, the AHP‐SOM algorithm [63], mNODE model [70], and the RF model [71] may conceptually constitute a future multi‐stage analytical framework spanning candidate screening, metabolic dynamics prediction, and microbial classification. Specifically, the AHP‐SOM approach can be used to identify potential bioactive components within natural systems; the mNODE model can simulate metabolic processes and generate dynamic predictions under specific dietary conditions; and the TCG‐RF (Twin Competitive Guild–Random Forest) framework can classify microbial communities and provide references for potential intervention design strategies. However, this proposed integration has not yet undergone experimental exploration or systematic validation and should therefore be regarded as a conceptual analytical framework for future investigation rather than an established workflow. If systematically developed and evaluated, such an integrative strategy may provide a broader framework for translating dietary inputs into targeted modulation of the gut microbiome and ultimately support more precise host‐level intervention designs.

### Intestinal mucus layer regulates host barrier function and shapes immune homeostasis

As the first physical and immune barrier between the host and intestinal microbiota, the dynamic balance of the intestinal mucus layer is associated with intestinal microbiota, dietary intake, and genetic factors, and the disruption of this balance is closely linked to various intestinal and metabolic diseases. Traditional experimental methods can only capture the state of the mucus layer at specific time points, making it difficult to predict its dynamic evolution during dietary changes, microbial disturbances, or disease progression. In contrast, AI technology provides powerful support for in‐depth analysis of mucus barrier mechanisms, prediction of its imbalance risk, and development of intervention design strategies. Among the core colonizing bacteria of the mucus layer, *Akkermansia muciniphila* is a typical representative, and its role in the human body is dual. Moderate colonization of *Akkermansia* can maintain intestinal health, improve metabolic disorders, and inhibit inflammation, especially showing prominent performance in intervention designs that directly or indirectly alleviate metabolic diseases; however, under specific pathological conditions, *Akkermansia* can aggravate intestinal inflammation and cause excessive consumption of the mucus layer, which needs to be judged in combination with the individual intestinal microecological state [76, 77]. Microbial culture and molecular biology methods are difficult to accurately predict their colonization state and functional tendency at the individual level, while AI models have shown unique advantages in addressing this problem. Wu et al. [52] used AI models such as Random Forest (RF) and mNODE to successfully predict the colonization outcome of *Akkermansia* based on the baseline composition of in vitro human fecal microbial communities, achieving high/low binary classification colonization and quantitative prediction, as well as reproducing the bimodal distribution of its abundance. This observation suggests a potential future application for prospectively evaluating whether *Akkermansia* in the individual intestine is associated with a beneficial moderate colonization state or a potentially unfavorable excessive proliferation state. Although there are currently no direct experimental studies that use AI to predict the specific functional phenotypes of *Akkermansia*, existing evidence supports the possibility that AI‐assisted analytical frameworks may contribute to characterizing its context‐dependent “beneficial” or “detrimental” functional tendencies [78]. Nevertheless, such applications remain speculative and require further experimental validation before establishing predictive utility. Machine learning‐based analyses of microbiome datasets have shown that microbial features, including *Akkermansia*, can be used to differentiate between healthy and disease‐associated microbiota states, indicating that AI is capable of capturing its context‐specific functional roles. At the same time, the functional outcomes of *Akkermansia* are shaped by complex host‐microbe‐environment interactions, including strain‐specific characteristics and metabolic contexts [79]. These multidimensional dependencies make this species particularly suitable for AI‐driven multi‐omics modeling approaches aimed at predicting its functional phenotypes under different physiological and environmental conditions. To further achieve the leap from phenotypic prediction to mechanism analysis, ML methods have also been used to mine functional genes from MAGs data [80]. The improved Prodigal tool was used to predict small open reading frames in massive MAGs and prokaryotic genomes, and then the Macrel pipeline was used to screen candidate antimicrobial peptides (cAMPs) to exclude single‐sample‐specific sequences. After constructing the AMPSphere database, the antimicrobial activity of some cAMPs was cross‐evaluated by multiple tools, ultimately realizing functional gene mining. This process enables AI‐assisted inference linking genotype to putative phenotype. If genomic data from Akkermansia are incorporated into such analyses, it is expected to screen its functional genes based on the same process to further reveal its mechanism of action. Therefore, aiming at the dual functional heterogeneity of *Akkermansia*, AI may provide a possibility for targeted regulation and promotion of its probiotic properties by analyzing the functions of key genes.

Mucus barrier disruption facilitates colonization by enteropathogens such as Clostridium perfringens via genomic adaptation, driving various intestinal and systemic diseases [81]. In Crohn's disease (CD), mutations in the NOD2 gene lead to abnormal defensin secretion and thinning of the mucus layer [82]; in patients with irritable bowel syndrome (IBS), intestinal dysbiosis, reduced SCFAs levels, and abnormalities in pathways such as IL‐22 are common [83, 84]. A high‐fat diet (HFD) can disrupt intestinal tight junctions and the mucus layer by reducing SCFA‐producing microbiota, leading to metabolic endotoxemia [85], which drives chronic inflammation in adipose tissue and the liver, and promotes the development of insulin resistance [86], non‐alcoholic fatty liver disease (NAFLD), and obesity [87]. Recent evidence further demonstrates that excessive sugar‐sweetened beverage intake during pregnancy can alter gut microbial composition and SCFAs profiles, compromise intestinal barrier integrity, and promote oxidative‐inflammatory and metabolic disturbances, further supporting the diet–microbiota–host health connection [88]. These disease phenotypes essentially reflect the systemic consequences of mucus barrier imbalance. The value of AI in integrating multidimensional data for disease diagnosis and microbiota intervention design is closely linked to the wide spectrum of diseases caused by mucus layer disruption. Li et al. [7] used algorithms such as least absolute shrinkage and selection operator (Lasso) to input microbial abundance data from 16S or MAGs sequencing, performed single‐cohort and cross‐cohort validation, and improved accuracy by constructing a combined model incorporating multi‐cohort samples of non‐intestinal diseases. Their findings indicated that the intestinal microbiota can serve as an effective independent diagnostic tool and provided strategies for optimizing cross‐cohort performance. This study demonstrated that analyzing microbial profiles through AI may indirectly reflect the state of the intestinal mucus layer, thereby providing auxiliary diagnostic value for diseases associated with mucus layer defects, such as CD and IBS. In addition, by efficiently analyzing large‐scale datasets such as MAGs, AI technology can identify microbial characteristic profiles associated with health, inform the design and regulation of microbiome engineering, optimize microbial diversity, and restore microecological balance [51]. These effects may help regulate mucus layer dysfunction caused by intestinal dysbiosis and reduced SCFAs levels. Probiotics such as Lactobacillus rhamnosus GG alleviate inflammatory bowel disease (IBD) and IBS by strengthening the mucosal barrier, modulating microbiota and attenuating inflammation [89], and their metabolites, such as SCFAs, also play key roles in strengthening barrier function and regulating immunity. AI has further accelerated the innovative application of probiotics in health promotion and disease intervention design [51].

Building on these advances of AI in mucus layer‐related diagnosis and probiotic application, its functions have extended from diagnosis to design and simulation in terms of dynamic intervention design and informed optimization. AI is gradually acquiring the ability to simulate and optimize ecological systems: while ML models such as neural networks are flexible and powerful, they suffer from poor interpretability; models such as the Lotka‐Volterra (LV) equation provide a biological structural framework but face difficulties in parameter fitting in complex systems. The MBPert framework [90], which combines the improved LV equation with ML optimization, possesses both mechanistic modeling and data‐driven capabilities. It can infer microbial interactions from perturbation and time‐series data, predict dynamic changes, accurately restore microbial community interactions, and provide mechanistic support for the design and regulation of microbiome engineering, optimization of microbial diversity, and restoration of microecological balance. This framework can be used to simulate how microbial communities change after supplementing specific prebiotics or probiotics, and ultimately predict whether such changes can promote SCFAs production or enhance mucus barrier function. Similarly, in the field of probiotic engineering, Gherman et al. [91] employed ML surrogate models combined with whole‐cell models (WCMs) to accelerate the design of genome reduction in *Escherichia coli* (*E. coli*). Conventional WCMs are comprehensive and grounded in biochemical reactions principles but are computationally intensive; in contrast, ML surrogate models can rapidly learn and simulate their input–output relationships, substantially accelerating genome reduction design in model organisms such as *E. coli*. When applied to probiotics or engineered strains development, this strategy may enable the efficient design of beneficial strains capable of targeted colonization within the mucus layer.

From a systems integration perspective, it is worth exploring the future development of an AI analytical platform that incorporates multi‐dimensional modeling frameworks. If the predictive outputs of the MBPert framework regarding microbial community dynamics are used as inputs for the mNODE model, the capacity to simulate long‐term temporal trends may be further improved. Alternatively, key targets identified through functional gene mining could be integrated into a WCM assisted by surrogate modeling approaches, thereby enhancing the potential for the rational design of functionally optimized probiotics. On this basis, a conceptual closed iterative loop‐from AI‐based prediction, to experimental validation in animal models, and subsequently to data feedback for model refinement‐may progressively enhance both the predictive accuracy and interpretability of the system, ultimately advancing research on the intestinal mucus layer and gut immunity toward a more predictable and controllable framework. However, it should be emphasized that this integrated workflow has not yet been experimentally evaluated, and its regulatory controllability remains limited at this stage. These proposed combinations should be interpreted as hypothesis‐generating methodological perspectives rather than established analytical pipelines ready for practical exploration.

Despite the rapid expansion of AI applications in microbiome research, several fundamental limitations remain unaddressed. The high dimensionality of microbiome datasets paired with relatively small sample sizes significantly elevates overfitting risk and impairs model generalizability [92, 93]. Compounding this issue, the inherent sparsity and compositional characteristics of microbiome data create unique statistical hurdles, such as spurious correlations and unstable feature selection, that further erode model robustness [94, 95]. Adding another layer of complexity, extensive biological heterogeneity between individuals and study cohorts severely limits the reproducibility and cross‐cohort transferability of AI models developed from these datasets [96]. Perhaps the most consequential limitation is that nearly all existing machine learning methods identify associations rather than causal relationships, restricting their utility for mechanistic inference and reliable prediction of intervention design outcomes [97].

Importantly, the majority of existing AI frameworks remain fundamentally correlation‐driven and lack robust causal inference capability. Consequently, although these models are effective in identifying predictive associations, they cannot reliably determine whether observed relationships reflect true underlying biological mechanisms, thereby limiting their translational value in guiding precise intervention designs.

### Gut‐X axis signaling network integrates multi‐pathway information and systematically regulates host functions

Beyond research on the intestinal mucus layer, the gut‐X axis signaling network enables bidirectional information transmission between the intestine, the central nervous system (CNS), and other distal organs through multiple channels including barrier permeability, microbial metabolites, neural pathways, and immune pathways, thereby influencing the initiation and progression of diseases. High inter‐individual heterogeneity in microbial composition, metabolic capacity, and host susceptibility further contributes to substantial variation in gut‐brain axis‐associated disease manifestations and underlying molecular mechanisms. Microbial‐derived signals constitute key mediators of gut‐brain communication. Disruption of the intestinal barrier promotes the translocation of lipopolysaccharide (LPS) into the circulation, triggering systemic and central inflammatory cascades that aggravate β‐amyloid (Aβ) deposition and neuroinflammation in Alzheimer's disease (AD) and Parkinson's disease (PD) [98, 99, 100]. Likewise, environmental arsenic exposure disrupts intestinal microbial homeostasis by downregulating intestinal fat mass and obesity‐associated gene (FTO) expression, increasing RNA m6A methylation, and impairing barrier integrity. In particular, the reduced abundance of Desulfovibrio fairfieldensis decreases microbial hydrogen sulfide (H2S) production, thereby exacerbating neurobehavioral abnormalities, including motor coordination deficits and anxiety, through the gut‐brain axis [101]. Conversely, beneficial microbial metabolites also contribute to host regulation. AI‐assisted genome‐scale metabolic models (GEMs) identified the probiotic strain Lactobacillus reuteri (L. reuteri) IMB015, which produces γ‐aminobutyric acid (GABA) while consuming glutamate, thereby alleviating cerebral inflammation and behavioral disorders [102]. At the molecular level, machine learning analysis of 1.09 million metabolite‐receptor interactions further identified agmatine and its receptor CA3R as a protective signaling pair, and experimental validation demonstrated that agmatine regulates CA3R expression and phosphorylated tau protein, providing mechanistic evidence linking microbial metabolites to neurodegenerative progression [103]. Collectively, these microbial signals converge on multiple host pathways, including neuroinflammation, neurotransmitter regulation, blood‐brain barrier dysfunction, and immune signaling, ultimately contributing to neurodegenerative disorders such as AD and PD as well as environmentally induced neurobehavioral impairment. Recent advances in nanomedicine have also provided complementary therapeutic approaches for modulating inflammation associated with these pathways [104]. The complexity and heterogeneity of gut‐brain axis interactions make conventional analytical approaches insufficient for reconstructing these multilevel relationships. AI has therefore emerged as an enabling computational framework for integrating microbiome, metabolomic, imaging, and clinical datasets to decipher gut‐brain axis mechanisms. Multi‐omics approaches incorporating multimodal brain imaging together with blood, saliva, and cerebrospinal fluid metabolite profiling enable AI models to reconstruct the cascade from microbial signals to CNS responses, thereby facilitating systematic analysis of gut‐brain communication [105]. At the ecosystem level, Sizemore et al. [106] developed Q‐net, a digital twin model based on infant 16S rRNA datasets that captures temporal microbial community dynamics, estimates neurodevelopmental risk, and simulates the potential effects of fecal microbiota transplantation (FMT) and dietary interventions. For example, microbiota‐derived metabolites such as 5‐hydroxyindole‐3‐acetic acid directly promote infant growth and neurodevelopment, which can be modeled by digital twin platforms like Q‐net [107]. At the disease level, Wu et al. [108] established a microbiota‐related risk score from multiple glioma cohorts, demonstrating significant associations between microbiome‐derived features, immune infiltration, and therapeutic responses. Collectively, current AI applications primarily contribute to cross‐level association modeling and mechanistic hypothesis generation rather than establishing causality. Although individual findings, such as the agmatine‐CA3R signaling axis, have received experimental validation [103], most AI‐assisted gut–brain axis studies remain dependent on retrospective datasets and correlation‐driven inference. Furthermore, substantial heterogeneity in microbial composition, host susceptibility, and environmental exposure continues to constrain model generalizability. Future progress will therefore require longitudinal datasets, prospective validation, and mechanistic experimentation to support translational application. Similar analytical strategies may also be extended to other gut‐related organ axes, where bile acid metabolism acts as a conserved One Health mediator [109], and dietary microbes and prebiotics modulate osteoarthritis and rheumatoid arthritis progression [110] (Figure 1A–F).

**Figure 1:**
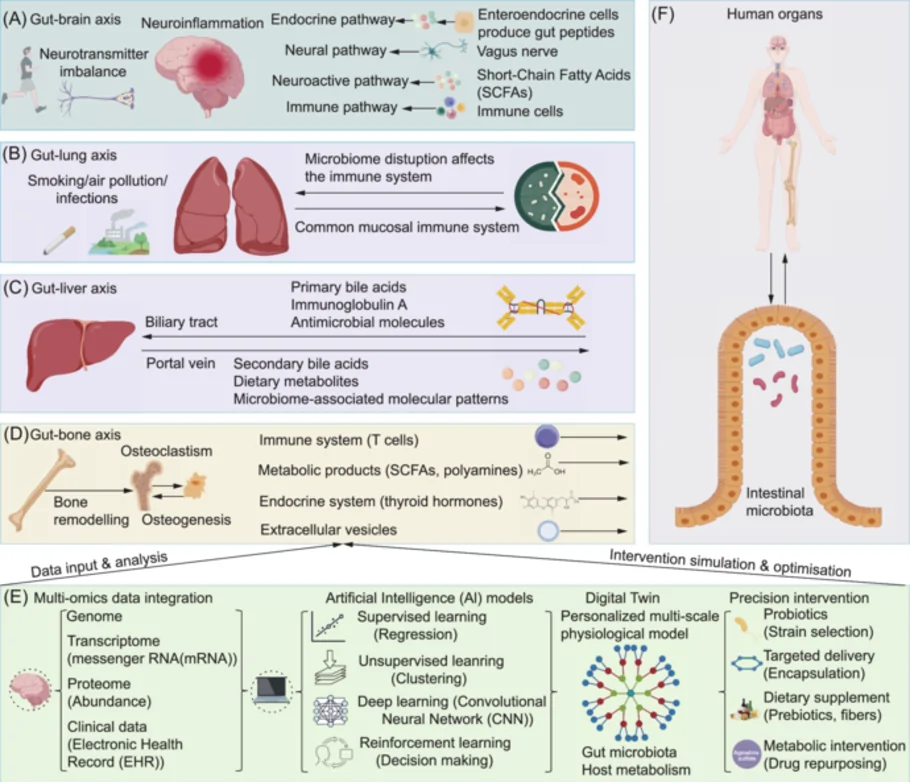
Artificial intelligence (AI)‐assisted integration of gut–organ axes. Major gut‐organ communication pathways, including the (A) gut‐brain, (B) gut‐lung, (C) gut‐liver, and (D) gut–bone axes, are illustrated within a diet–microbiota–host framework. These interactions involve neural, endocrine, immune, and metabolic signaling pathways through which the intestinal microbiota and microbial metabolites influence host physiology and organ function. Representative mechanisms include microbiota‐derived regulation of neurotransmitters and neuroinflammation in the gut–brain axis, immune and microbial interactions in the gut‐lung axis, bile acid and metabolite exchange in the gut–liver axis, and microbiota‐mediated regulation of bone remodeling through immune and endocrine signaling in the gut–bone axis. (E) AI is presented as an integrative layer that enables systems‐level analysis and interpretation of microbiota–host interactions. AI‐assisted workflows incorporate multi‐omics data integration, including genomic, transcriptomic, proteomic, and clinical data, to construct comprehensive biological profiles. Machine learning (ML) approaches, including supervised learning, unsupervised learning, deep learning (DL), and reinforcement learning, support pattern recognition, prediction, and decision‐making. AI further facilitates the development of digital twin models for personalized simulation of the intestinal microbiota, host metabolism, immune responses, and inter‐organ interactions, and enables precision interventions, including probiotic optimization, targeted delivery systems, dietary supplementation, and metabolic regulation strategies. (F) Conceptual overview of the bidirectional interactions between the intestinal microbiota and multiple host organs, representing the systems‐level biological foundation for AI‐driven modeling, simulation, and intervention optimization within the diet‐microbiota–host framework.

Building upon the above biological framework, complementary AI models can be integrated to improve mechanistic interpretation and translational prediction across different analytical levels of the gut‐X axis. Q‐net excels at simulating dynamic changes in microbial community structure and associated host‐level health outcomes at the ecosystem scale; however, its interpretation of specific metabolic functions remains relatively indirect. In contrast, bacterial GEMs can accurately predict the metabolite output of specific bacterial strains. The combination of these two models enables functional verification based on structural prediction: when Q‐net identifies a state of microbial dysbiosis, GEMs can subsequently be employed to screen probiotic candidates capable of compensating for key metabolite deficiencies, thereby facilitating the design of more targeted microbial intervention design strategies. On the other hand, ML‐driven targets such as agmatine‐CA3R can identify specific molecular pathways but lack the evaluation of clinical translational value. In contrast, the MRS can be correlated with glioma immune infiltration characteristics and treatment responses. The combination of these two approaches can not only achieve mechanistic interpretation but also further complete clinical validation: after discovering the protective effect of agmatine, a prognostic model based on intestinal metabolite profiles can be constructed using a similar approach to MRS, so as to verify the universality and clinical significance of this pathway in patient populations. Despite the increasing application of AI in gut‐X axis studies, substantial heterogeneity in microbial composition, host susceptibility, and environmental exposure continues to constrain model generalizability. Moreover, most multimodal frameworks remain correlation‐driven and require stronger mechanistic validation to support translational application. In addition, while the preceding sections primarily focus on mechanistic insights derived from human studies and experimental model systems, an equally important issue concerns how microbiota‐host interactions differ across species and biological contexts.

### Intestinal microbiota of animals with different digestive physiological types exhibit differential adaptive strategies

While the preceding sections primarily focus on biological mechanisms within the human intestinal microbiota, the implications of this framework extend well beyond a single host system. In this context, the diet‐AI–microbiota–host interaction serves as the central conceptual framework, whereas concepts such as “One Health” and cross‐species health are regarded as downstream application scenarios in which this framework can be further explored, extended, and evaluated across diverse biological and ecological systems.

Under the “One Health” framework, the microbial adaptation mechanisms of livestock and humans hold equal research significance. To place microbiota–host interactions within a broader biological context, it is important to consider interspecies differences in digestive strategies. Different animal groups, including monogastric animals and ruminants, possess distinct gastrointestinal structures and microbial ecosystems, which consequently influence the metabolism of dietary components as well as the transmission and regulation of microbial‐derived signals within the host. Human microbiota‐associated animal models provide an important experimental bridge by enabling the transplantation of human‐derived microbial communities into animal hosts, thereby allowing controlled investigations of diet–microbiota–host interactions while maintaining translational relevance to human biology [111]. In particular, pig models exhibit greater physiological and gastrointestinal similarity to humans, which may improve the translational reliability of microbiome–diet interaction studies across species [112]. From a computational perspective, recent advances in AI, particularly in transfer learning methodologies, enable models trained within one microbial or host context to be adapted to alternative systems with limited available data, thereby providing a potential framework for cross‐species microbiome integration [113]. Transfer learning‐based frameworks such as EXPERT have demonstrated the capacity to generalize microbial community classification across different environmental contexts, suggesting that similar strategies may also be extended to cross‐host predictive applications [114]. Cross‐species integration is not merely a conceptual objective, but is increasingly emerging as a data‐driven research paradigm in which AI facilitates the transfer and translation of microbiome‐derived insights across diverse biological systems. Nevertheless, cross‐species transfer of AI models based on gut microbiome information remains constrained by biological and methodological heterogeneity. Differences in physiological characteristics, particularly gastrointestinal architecture across hosts, may lead to variations in nutrient utilization patterns and microbial ecological niches, thereby limiting the direct transferability of models across humans, pigs, ruminants, and other species [115]. In addition, variation in diet matrices, microbial strain‐level functional specificity, and host‐dependent microbial colonization processes may further reduce model generalizability across different populations and environmental contexts [116, 117]. Meanwhile, cohort‐specific factors, including differences in sequencing strategies, sample composition, and data preprocessing pipelines, may introduce additional bias and increase the risk of negative transfer [118]. Therefore, transfer learning should currently be regarded as a promising yet exploratory strategy, and its broader application in cross‐species microbiome prediction will require the establishment of standardized multi‐species datasets together with systematic biological validation.

The intestinal microbiota of livestock is a core factor influencing digestion, immunity, and production performance, and its informed optimization has become pivotal for healthy breeding. However, the complexity and dynamism of this system pose substantial challenges to research and application. AI has been introduced as a powerful analytical and predictive tool in animal microbiology studies (Figure 2). In terms of inherent differences in microbial systems, ruminants and monogastric animals possess distinct fermentation systems: the former relies on a synergistic microbial network dominated by *Bacteroidetes* and *Firmicutes* in the multi‐chambered stomach for microbial fermentation of plant fibers [119, 120], with methanogenic archaea such as *Methanobrevibacter* contributing to methane production and overall fermentation system stability [120, 121]; the latter primarily undergoes hindgut fermentation, and their microbial composition and functions vary by species. Meanwhile, the microbiota is highly sensitive to factors such as animal diets: high‐fiber forage increases the abundance of fiber‐degrading bacteria, while high‐grain diets promote the proliferation of starch‐degrading bacteria such as *Succinivibrio* [120]; breed differences significantly alter colonic host‐microbe interactions in pigs [122]; high enteric starch diets can induce hindgut microbial dysbiosis, whereas low‐starch diets maintain Bacteroidetes‐dominated homeostasis [123], and inappropriate diets may even trigger hindgut microbial imbalance. However, the structure and function of microbial communities are complexly influenced by multiple factors such as diet, environment, and host genotype, making it difficult to accurately predict and regulate their dynamic changes through traditional methods. AI technology has emerged as a key tool bridging macro‐phenotypes and micro‐mechanisms, thereby providing new strategies for precision livestock farming (PLF) and health management. Maaskant et al. [8] developed a fecal image analysis technology based on DL and ML for rhesus macaques, integrating image data with MAGs data. This technology enables rapid prediction of 16 bacterial genera (including butyrate‐producing genera) and butyrate‐producing functional microbial communities (AUC = 0.75, area under the receiver operating characteristic curve). Through non‐invasive rapid detection, the technology shortens the originally time‐consuming MAGs analysis process to the minute level and reduces costs by over 90%. This method demonstrates the enormous potential of AI in achieving rapid and low‐cost monitoring of animal microbiota, potentially allowing farmers to timely evaluate the intestinal health status of animals and adjust dietary formulas. In practical applications, the capability of AI in multimodal data integration and phenotypic recognition has also provided important technical support for the investigation of diseases associated with microbial dysbiosis. Wen et al. [124] employed a multi‐model fusion strategy for the diagnosis of porcine gastrointestinal infectious diseases, incorporating processes. They used ChatGPT‐assisted text augmentation and image enhancement to augment porcine gastrointestinal infectious disease case data, adopted multi‐scale text convolutional neural networks (TextCNN) to extract semantic features from case texts, utilized Mask Region‐Based Convolutional Neural Network (Mask R‐CNN) to segment small intestinal lesion areas, and then combined convolutional neural networks (CNN) to classify lesion images. This integrated framework substantially improved disease recognition efficiency and offered a technical approach for indirectly reflecting intestinal microecological states through host phenotypic characteristics. Although its connection to the “diet–microbiota–host” axis remains relatively indirect, this study nevertheless provides a valuable reference for the integration and application of multi‐source information in complex biological systems, particularly in addressing the practical challenge of limited availability of high‐quality annotated datasets.

**Figure 2:**
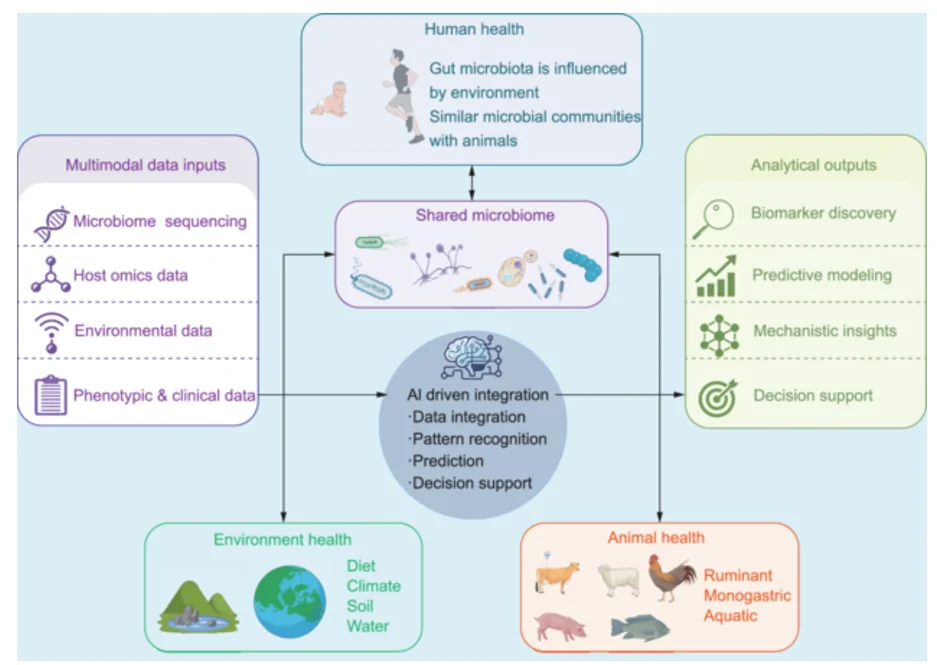
Cross‐species extension of the four‐dimensional framework. Schematic representation of the interconnected relationships among human, animal, and environmental health within a One Health framework. AI supports the cross‐species integration and interpretation of host–microbiota–environment interactions, thereby facilitating the identification of shared mechanisms, risks, and intervention opportunities.

Fortunately, existing models exhibit complementarity, with the study by Wen et al. [124] serving as a paradigmatic example. TextCNN is specifically designed for sentence‐level text feature extraction, enabling the extraction of key symptom descriptions and medical histories from case reports, while Mask R‐CNN and CNN are inclined toward image processing, facilitating the identification of lesion regions from medical images. By combining these two types of models‐unifying, mapping, and concatenating the text features extracted by TextCNN with the image features extracted by Mask R‐CNN‐deep fusion of multi‐modal information can be achieved. This integration allows for the synthesis of clinical descriptions and visual evidence to generate more reliable comprehensive diagnoses. Building on the complementarity of existing models, future research could utilize CNN for visual data processing, natural language processing (NLP) for text report analysis, and temporal models such as long short‐term memory (LSTM) or graph neural networks (GNNs) for exploring dynamic interaction relationships within microbial communities. Ultimately, this hypothetical workflow for future investigation would model potential long‐term impacts of different dietary or management strategies on microbial ecosystems and animal health, providing farmers with optimal intervention design schemes. Extending this framework to non‐human systems represents not merely a shift in research focus but also a critical assessment of its generalizability across biological contexts. The intestinal microbiota‐mediated processing of dietary inputs constitutes a conserved biological principle observed across multiple species, thereby providing a conceptual and mechanistic foundation for cross‐species integration within the One Health framework. While AI provides opportunities for cross‐species microbiome integration, physiological and ecological differences among hosts remain major barriers to model transferability. Future progress will depend on standardized datasets and adaptive learning frameworks capable of preserving biological specificity across systems.

## 3 ARTIFICIAL INTELLIGENCE INTEGRATES MULTI‐OMICS INFORMATION AND RECONSTRUCTS MICROBIAL REGULATION STRATEGIES

### Artificial intelligence integrates multi‐omics data and constructs community metabolic models

The diversity of the intestinal microbiota exerts a profound influence on human health, and its dysbiosis has been demonstrated to be closely associated with various metabolic diseases such as diabetes mellitus and AD. Accurate interpretation and modulation of the intestinal microbiota have emerged as frontier topics in contemporary scientific research. Multi‐omics approaches, including MAGs, metatranscriptomics, metabolomics, and metaproteomics, are capable of depicting the complexity of the intestinal microbiota ecosystem. These tools hold significant clinical application potential: MAGs and metaproteomic analyses yield detailed profiles of microbial composition and functional characteristics, while metatranscriptomic and metabolomic methods add dynamic insights by uncovering microbial activities and biochemical crosstalk between the microbiota and the host. MAGs analysis can be categorized into four dimensions [125, 126, 127, 128, 129, 130, 131, 132, 133, 134, 135, 136, 137, 138, 139, 140, 141, 142, 143, 229] (Table 2), namely the one‐dimensional (1D) compositional level, two‐dimensional (2D) genetic diversity level, three‐dimensional (3D) structural biology level, and four‐dimensional (4D) spatiotemporal dynamic level [144]. However, the significant discrepancies in scale, dimension, and other aspects among multi‐omics datasets pose computational and methodological challenges in information integration, analysis, and mining. This makes it difficult to effectively integrate data and derive conclusions with clinical value, necessitating more efficient analytical frameworks.

**Table 2:** Four‐dimensional analytical framework in metagenomics.

Dimension	Application	AI model	Function	References
1D	Species identification and genome reconstruction	VAMB	Genome reconstruction from co‐abundance and sequence features	[127]
		SemiBin	Metagenome‐Assembled Genomes (MAGs) binning via contrastive learning	[128]
	Taxonomic classification	BERTax	DNA‐based taxonomic classification	[129]
	MAGs‐based taxonomic profiling	MetaPhlAn 4	Species‐level microbial profiling	[130]
	Disease model construction	DeepCoDA	A DL framework for analyzing relative microbial abundance data to model disease‐associated microbial signatures	[131]
	Clinical diagnostic modeling	eXtreme Gradient Boosting (XGBoost) (XGB‐IBD10)	Develops classification models based on the XGBoost algorithm to distinguish patients with Inflammatory Bowel Disease (IBD) from healthy controls and to identify disease subtypes	[132]
	Biomarker discovery	RF with explainable AI (XAI)	Identifies Colorectal Cancer (CRC)‐associated microbial biomarkers, including *Fusobacterium nucleatum* and *Peptostreptococcus* spp	[126, 133]
2D	Variant detection	DeepVariant	Reformulates variant calling as an image classification task and employs CNNs to identify sequence variants in microbial genomes	[134]
3D	Protein structure and function prediction	HMD‐ARG	Uses hierarchical multi‐task DL to predict antibiotic resistance genes and their associated properties	[135]
		AlphaFold	Predicts three‐dimensional protein structures directly from amino acid sequences using DL models	[136]
		Foldseek	Infers protein function through rapid structural similarity searches against known protein structures	[137]
	Protein generation	ProGen	Trains Transformer‐based Large Language Models (LLM) to predict amino acid sequences and generate functional proteins	[138]
	Model interpretability	SHapley Additive exPlanations (SHAP)	Quantifies the contribution of individual features to model predictions, improving the interpretability of AI‐driven analyses	[229]
4D	Spatiotemporal dynamic model	MaPS‐seq	Fixes microbial samples within a gel matrix to resolve and analyze the spatial organization of microbial communities	[139]
		MCSPACE	Models spatiotemporal dynamics of microbial communities using data from single‐cell sequencing, organ‐on‐chip systems, and related platforms	[140]
	Multi‐omics integration and prediction	BioMapAI	Integrates intestinal MAGs with plasma metabolomic profiles to predict disease severity	[62]
	Therapeutic optimization	Longitudinal MAGs sequencing	Evaluates the efficacy of Fecal Microbiota Transplantation (FMT) in patients with Ulcerative Colitis (UC) and identifies treatment‐associated bacterial strains	[141]
	Risk prediction	Random survival forest models based on MAG data	Constructs microbiome‐based risk scores to predict the risk of Crohn's Disease (CD)	[125, 142]

Against this backdrop, the multi‐dimensional data fusion capability of AI has emerged as a crucial technical pillar for multi‐omics data research. Its application in multi‐omics integration is steadily advancing the elucidation of disease mechanisms, the discovery of diagnostic biomarkers, and the optimization of clinical intervention design strategies. In recent years, GEMs, as mathematical tools integrating genomic, biochemical, and physiological data, have enabled the prediction of microbial metabolic behaviors. It should be emphasized that these predictions are primarily derived from statistical associations rather than confirmed causal relationships, and therefore still require rigorous experimental and mechanistic validation. Ding et al. [145] developed an AI‐assisted tool named BEAUT to mine bile acid metabolic enzymes and supports the construction of microbiome metabolic resources. Tools including BEAUT and BioMGCore support microbiome metabolic resource construction and GEMs development [146]. This provides new tools and targets for microbial metabolism research and disease intervention design. Nevertheless, a single GEM can only characterize the metabolic capacity of an individual bacterial strain. To conduct more comprehensive and systematic investigations on microbial communities, community‐level metabolic models (Co‐GEMs) have been developed by integrating multiple single‐strain models and MAGs data. This integration has demonstrated enormous potential in intestinal microbiota research [125], as it can predict interactions between different strains and the overall metabolic capacity of the community, reveal the functional roles of various species within the community, and uncover potential mechanisms linking these species to host diseases. Consequently, Co‐GEMs exhibit promising application prospects in fields such as precision medicine and personalized nutrition. AI‐driven microbiome matching frameworks have further expanded the application scope of precision microbiota intervention design. Su et al. developed MOZAIC, a deep learning framework capable of predicting donor‐recipient microbiome convergence after FMT by integrating multi‐kingdom microbial and functional features, thereby significantly improving predicted clinical response rates [147]. Meanwhile, the metaGEM tool enables direct generation of GEMs from MAGs data [148, 149, 150]. The pan‐model approach Pan‐Draft [149], incorporated into the gapseq pipeline, enables the construction of species‐representative GEMs by comparing MAGs clustered at the species level and quantifying the frequency of non‐redundant metabolic reactions in the pan‐reactome. It also addresses the issues of incompleteness and contamination in individual MAGs, improving the structural quality and predictive accuracy of species‐level GEMs. Additionally, Pan‐Draft improves species‐level GEM completeness. At the structural omics level, the intestinal microbiota protein structures deposited in the GMPS database [151] provide structural annotations supporting metabolic interpretation. Additionally, personalized intestinal microbiota community‐level metabolic models (Co‐GEMs) serve to characterize the metabolic signatures of the intestinal microbiota and identify key disease biomarkers. The MIGRENE toolkit utilizes gene catalogs and MAGs‐based species pan‐genomes to enable the entire process from constructing context‐specific GEMs to generating personalized Co‐GEMs, thereby allowing the metabolic characteristics of an individual's intestinal microbiota to be described [152]. The MIGRENE enables personalized community metabolic reconstruction [152]. This demonstrates its utility in deciphering microbe‐host interactions as well as its potential for personalized medicine and disease management. Beura et al. [153] constructed Co‐GEMs for healthy individuals and patients with T2DM, revealing that metabolic fluxes of metabolites such as SCFAs and vitamin B5 were significantly reduced in the Co‐GEMs of T2DM patients. They also found that decreased catabolism of branched‐chain amino acids and aromatic amino acids may serve as a unique biomarker for T2DM. Martinelli et al. [154] demonstrated that the formate secretion capacity of Co‐GEMs from AD patients was impaired. Comparison with urine metabolomic data confirmed that formate could be a potential early biomarker for AD.

Owing to its inter‐individual variability and plasticity, the intestinal microbiota serves as a modifiable target for precision medicine. However, heterogeneity across multiple cohorts remains a major bottleneck in microbial biomarker research. GMrepo [155], the largest AI‐powered human intestinal microbiota database to date, integrates a vast array of samples and phenotypes while introducing, for the first time, a large language model (LLM)‐assisted data filtering mechanism. Databases including GMrepo and FoodMicroDB support standardized microbiome data integration with LLM‐assisted curation [156]. The Microbial Consistency Index proposed by this database facilitates the systematic quantification of the robustness of microbial biomarkers across different studies, thereby significantly enhancing the reproducibility of analyses across research projects and populations. Given the ongoing expansion in sample sizes and the increasing diversity of acquired datasets, the integration of multi‐omics technologies emerges as a promising strategy. Incorporating more advanced AI or ML techniques into microbiome and multi‐omics research can identify previously undetectable patterns, which can be applied to multiple aspects of precision medicine exploration.

Multi‐omics integration is further advancing model upgrading. Condition‐specific community‐scale metabolic models (CoCo‐GEMs), incorporating metatranscriptomic data, dynamically delineate community metabolic activity and provide a more granular framework for the design of precision intervention designs [157, 158]. Via databases such as AGORA, species‐level GEMs can be retrieved, and subsequently, species abundances and metabolic interactions are integrated through community model frameworks like MICOM [159]. This facilitates accurate prediction of key metabolites such as SCFAs [159, 160, 161], and model optimization can be tailored according to specific research objectives to generate condition‐specific community models. Tools like CoCo‐GEMs have advanced by integrating metatranscriptomic data, and future work could attempt the full integration of metabolomics.

These tools operate at different analytical layers rather than representing equivalent AI systems. Specifically, BEAUT and MOZAIC primarily function as AI‐driven predictive frameworks, whereas metaGEM, Pan‐Draft, MIGRENE, Co‐GEMs, and CoCo‐GEMs focus on metabolic reconstruction and simulation. GMPS serves as a structural resource, and MICOM enables community‐level metabolic inference. Their integration forms a hierarchical workflow spanning data acquisition, metabolic reconstruction, interaction prediction, and personalized intervention design. To improve conceptual clarity and distinguish AI algorithms from metabolic reconstruction tools, databases, and analytical pipelines, representative frameworks involved in multi‐omics integration and GEM‐based microbiome modeling are summarized [125, 145, 147, 148, 149, 150, 151, 152, 153, 154, 155, 157, 158, 159, 160, 161] (Table 3).

**Table 3:** Representative AI‐assisted multi‐omics and genome‐scale metabolic models frameworks for microbiome metabolic modeling and precision intervention design.

Tool/Framework	Input data	Output	AI/modeling strategy	Primary biological objective	Major limitation	References
BEAUT	Gut microbial genomes, MAGs, bile acid metabolic genes	Human Gut Bile Acid Metabolic Enzyme (HGBME) database; candidate enzymes and metabolic targets	AI‐assisted enzyme annotation and metabolic inference	Bile acid enzyme discovery	Limited mechanistic causality; relies on annotation completeness and metabolic inference accuracy	[145]
Co‐GEMs	Multiple strain‐level GEMs, MAGs, species abundance	Community metabolic fluxes, metabolite production, microbial interaction network	Constraint‐based metabolic modeling	Microbial metabolic interaction	Static assumptions; limited temporal dynamics and environmental adaptation	[125]
MOZAIC	Multi‐kingdom microbiome profiles, microbial functional features, donor‐recipient FMT data	Predicted donor‐recipient microbiome convergence and response probability	Deep learning‐based microbiome matching	FMT matching	Limited interpretability and dependence on large matched cohorts	[147]
metaGEM	MAGs data	Species‐level genome‐scale metabolic models (GEMs)	Automated metabolic reconstruction workflow	Metabolic reconstruction	Model quality strongly depends on MAG completeness	[148, 149, 150]
Pan‐Draft	Species‐clustered MAGs, pan‐reactome information	Species‐representative GEMs and reaction catalog	Comparative metabolic reconstruction and gap‐filling	GEM optimization	Limited performance for low‐abundance species and unknown reactions	[149]
GMPS	Microbial protein structures and genomic annotations	Protein functional annotation and metabolic pathway reconstruction	Structural bioinformatics and protein modeling	Functional annotation enhancement	Structural prediction does not directly quantify metabolic activity	[151]
MIGRENE	Gene catalog, MAG‐based species pan‐genomes, host‐associated microbiome data	Context‐specific GEMs and personalized Co‐GEMs	Multi‐layer metabolic reconstruction framework	Personalized metabolic modeling	Limited longitudinal prediction and cross‐cohort transferability	[152]
Co‐GEM application (Type 2 Diabetes Mellitus (T2DM))	Personalized microbiome composition and GEMs	Disease‐associated metabolic flux changes	Community metabolic simulation	T2DM metabolic profiling	Association‐based; not causal	[153]
Co‐GEM application (Alzheimer's Disease (AD))	AD‐associated microbiome profiles	Predicted metabolic biomarkers	Community flux balance analysis	AD biomarker identification	Biomarker transferability remains uncertain	[154]
GMrepo v3	Human microbiome datasets and phenotype metadata	Harmonized microbial resource and Microbial Consistency Index (MCI)	LLM‐assisted data filtering and database integration	Cross‐cohort harmonization	Dependent on source data quality and annotation consistency	[155]
CoCo‐GEMs	Co‐GEMs + metatranscriptomics	Dynamic community metabolic states	Transcriptome‐constrained metabolic simulation	Dynamic metabolic simulation	Transcript abundance may not reflect actual metabolic flux	[157, 158]
MICOM	Species abundance, species‐level GEMs	Community metabolite prediction (Short‐Chain Fatty Acids (SCFAs), etc.)	Constraint‐based community optimization	Metabolite prediction	Sensitive to abundance estimation and model assumptions	[159, 160, 161]

Beyond microbiome‐ and host‐derived omics information, food processing is another important determinant of diet–microbiota interactions, that is, often overlooked. Existing AI models generally use nutrient composition or dietary intake as primary inputs, while processing‐induced changes in food properties and their influence on microbial substrate availability are rarely considered. In practice, processing methods such as thermal treatment, fermentation, and extrusion can alter nutrient release dynamics and bioaccessibility [162, 163], thereby affecting the composition of compounds that reach the intestine and become available for microbial utilization, ultimately shaping microbial community structure and metabolic responses [162, 164]. For example, mechanical and thermal processing can disrupt plant cell wall structures, thereby increasing the bioaccessibility of carotenoids and other bioactive compounds, whereas excessive refinement may accelerate digestion and consequently reduce the delivery of fermentable substrates to the colon [162, 164].

Future AI frameworks may incorporate food processing characteristics and bioavailability‐related parameters into multi‐omics integration pipelines [165]. Combined with digestion simulation data, metabolomic information, and Co‐GEMs, such approaches could provide more accurate estimation of microbial‐accessible substrates and facilitate the transition from current composition‐based prediction toward a more mechanism‐driven diet–microbiota–host research paradigm, thereby supporting precision nutrition and functional food design [165]. In particular, AI models could be developed to quantitatively predict how specific processing conditions modulate the bioaccessibility of nutrients and non‐nutrient bioactives by integrating data derived from in vitro digestion systems and food microstructure characterization, thereby enabling more accurate simulation of diet–microbiota interactions [164]. Despite their strong integrative capability, AI‐driven multi‐omics frameworks remain sensitive to batch effects, inconsistent preprocessing pipelines, and cohort heterogeneity. Furthermore, community metabolic predictions frequently rely on inferred interactions and therefore still require experimental confirmation before practical exploration.

### Artificial intelligence explores microbial functions and predicts interaction networks

With the advancement of gene sequencing technologies and declining sequencing costs, large‐scale microbiome studies have expanded substantially. Traditional statistical methods are ill‐equipped to process the massive and sparse microbiome datasets, making machine learning (ML) the preferred approach for analyzing microbiome data in complex contexts. Within microbiome research, dissecting microbial functions and predicting their interaction networks represent core objectives for revealing the ecological principles of microbial communities and developing targeted therapeutic strategies. Knowledge graphs, by virtue of their capacity to integrate multi‐source heterogeneous data and uncover complex interconnections, have provided breakthrough research ideas and methods to this domain, demonstrating tremendous application potential [166]. ML assumes a pivotal role in three key areas: the classification and prediction of microbial taxa, disease prediction, and the association between microbes and drugs.

Numerous innovative algorithms have been proposed and applied for microbiome data analysis. Wang et al. [167] addressed single‐omics scenarios and developed a novel DL prediction method named M Deep. Through the construction of convolutional layers to simulate taxonomic hierarchies, this method captures phylogenetic correlations among microbial species across all hierarchical levels, enabling the prediction of continuous and binary outcomes. Reiman et al. [168] prioritized structured sample processing and developed a CNN‐based DL tool called PopPhy‐CNN. This tool leverages CNN to identify locally similar microbial patterns within taxonomic trees and assess the importance of each taxon in predicting host phenotypes. Furthermore, the variational Bayesian microbiome multi‐omics (VBayesMM) algorithm, designed for multi‐omics data, adopts a Bayesian neural network architecture coupled with a spike‐and‐slab prior. It not only enables automatic selection of microbial features but also processes 16S rRNA and MAGs sequencing data based on a variational inference optimization strategy, while quantifying microbe‐metabolite co‐occurrence relationships via conditional probability matrices [169]. This innovative architectural design retains the advantage of neural networks in handling nonlinear relationships and delivers probabilistic interpretations through the Bayesian framework. Collectively, these three studies demonstrate that phylogenetic or taxonomic structural information of microbial taxa serves as a key prior for improving the performance of prediction models, and DL techniques can capture such structured information more efficiently than traditional ML methods, facilitating the performance enhancement of structured information‐driven microbial prediction models. However, existing models remain hampered by common limitations that impede their generalizability. Going forward, the Bayesian feature selection of VBayesMM could be integrated with the dynamic modeling capability of MDSINE2, or multi‐omics data, such as phylogenetics and host genomics, could be integrated into the models. A variety of artificial intelligence models with distinct functions have been employed in the research on diet–microbiota–host [63, 70, 71, 90, 99, 104, 167, 168, 169] (Table 4). Such integrations would further boost the robustness and predictive power of these models whilst preserving interpretability. Exploring the associations between such modeling approaches and host characteristics is crucial for understanding their impacts on various diseases. Although shotgun MAGs sequencing technology has yielded vast volumes of microbiome data, these datasets exhibit sparsity, noise, and high feature dimensionality, making their analysis highly challenging. Knowledge graph methodologies offer a promising strategy for mitigating these challenges, primarily through network construction and analysis. Notably, the AEPMA framework deserves special attention: it constructs a Peptide‐Microbe‐Disease Heterogeneous Network (PMDHAN) encompassing various peptides, microbes, and diseases. By integrating information from multiple databases, AEPMA facilitates accurate prediction of associations between antimicrobial peptides (AMPs) and microbes [46]. Beyond AEPMA, other AI models have also demonstrated robust capabilities in predicting microbe‐disease associations [170]. MIOSTONE is a neural network model specifically designed for microbe‐disease association prediction, serving as a natural bridge from changes in microbial taxa abundance to alterations in phenotypic features. Accordingly, it can be regarded as a complement and extension of AEPMA. Specifically, it extends AEPMA's AMP–microbe prediction scope to microbiome‐disease associations, leveraging a stochastic gating mechanism and large‐sample pre‐training to boost small‐sample performance. Furthermore, MIOSTONE mitigates AEPMA's limitations, including poor adaptability to data‐imbalanced settings, inadequate cross‐dataset generalization ability, and limitations in taxon‐level association modeling. Furthermore, it holds considerable utility for the management and treatment of diseases such as autism spectrum disorder (ASD), AD, Graves' disease, PD, and IBD.

**Table 4:** Representative AI analytical strategies aligned with biological questions.

Model	Input modality	Metric	Representative performance	Biological scale	Typical task	References
Analytical Hierarchy Process‐Self‐Organizing Map (AHP‐SOM)	Chemical/bioactive compounds	Accuracy/consistency	Improved decision consistency	Molecular	State evaluation	[63]
mNODE	Multi‐omics + time‐series	*R* ^2^/Spearman	*R* ^2^ ≈ 0.60–0.80	Microbiome/metabolic dynamics	Metabolite prediction	[70]
RF/TCG‐RF	Microbiome abundance	AUC	0.82–0.94	Community	Microbiome classification	[71]
MBPert	Time‐series microbiome data	RMSE/Pearson	*r* ≈ 0.7–0.9	Ecosystem/interaction network	Community perturbation	[90]
Q‐net	Longitudinal microbiome data	Forecast error	Error reduction 20–40%	System/host outcome	Digital twin	[99]
GEMs	Genomic + metabolic network	Precision/Recall	>80% reaction recovery	Molecular/metabolic	Metabolic reconstruction	[148]
M Deep	Microbiome data	Accuracy	~0.82	Community	Disease prediction	[167]
PopPhy‐CNN	Phylogenetic structure	ROC‐AUC	0.68–0.94	Community	Host phenotype prediction	[168]
VBayesMM	Multi‐omics	AUC	0.85–0.92	Multi‐scale	Multi‐omics integration	[169]

Identifying microbe–drug associations is conducive to enhancing understanding and accelerating rational drug development. With the rapid expansion of genomic and pharmacological datasets, computational methods are required to identify such associations. To date, the basic assumption of various predictive computational methods is that if a drug is associated with a microbe, structurally analogous drugs are also likely to be associated with the same microbial species. GCNMDA employs an integrated framework that combines conditional random fields (CRFs) and graph convolutional networks (GCNs), constructing a heterogeneous network and extracting node features to achieve association prediction to a limited extent. However, such computational approaches often exhibit a strong reliance on known associations in the network, resulting in limited predictive capability for low‐ prevalence or uncharacterized drugs and microbes [171]. Furthermore, the prediction accuracy of these methods is unstable, given that drug structural similarity does not always accurately reflect their interaction relationships with microbes. To mitigate the aforementioned limitations, the AAHLDMA model utilizes more abundant biological information to derive robust representations of drugs and microbes, deploys graph autoencoders to learn the latent representations of drugs and microbes [172], and innovatively introduces the concept of “bridge nodes” to connect drugs and microbes, thereby constructing a learnable drug‐microbe interaction network. Experimental validations show that the AAHLDMA model performs excellently on multiple benchmark datasets, with an average AUC value of 0.9581 and an area under the precision‐recall curve (AUPR) value of 0.9587, which is significantly superior to other existing methods. Currently, all models have a high dependence on data: the construction of the PMDHAN network in AEPMA relies on known association data from databases including DBAASP and Disbiome; the taxonomic hierarchy modeling in MIOSTONE depends on taxonomic annotations from the GTDB database. Any data omissions or classification errors within these databases will directly affect model performance. Song et al. [173] employed artificial neural networks (ANNs) as an alternative model for flux balance analysis, reducing the computational time required for dynamic simulation of microbial metabolic processes by approximately 1000‐fold and effectively mitigating the numerical instability problem that occurs when traditional methods simulate metabolic switching, thereby improving tolerance to data quality at the computational level. Beyond models tailored specifically for drug‐microbe associations prediction, various DL architectures also play important roles in related fields. CNNs are suitable for processing spatial data, recurrent neural networks (RNNs) excel at deciphering temporal patterns, autoencoders can achieve data dimensionality reduction [174], and GNNs are capable of simulating microbial interaction networks, providing a theoretical framework for synthetic microbiome design [10]. AI‐aided protein engineering can also enable the accurate prediction of protein structures and functions. The study by Chakraborty et al. [15] provided a protein‐level supplement for elucidating the mechanisms underlying microbe‐drug associations: they generated independent modules (iModulons) using optimal independent component analysis (Optica), then combined AlphaFold to complete the functional annotation and validation of hypothetical proteins, addressing the shortcomings of association prediction models such as GCNMDA in terms of mechanism interpretation. Additionally, this approach can also inform the design of intelligent screening libraries [143, 166]. The machine learning (ML)‐informed mutation library discovered by Saito et al. [143] provided a technical extension for the optimization of detection tools; the model iteration framework they developed can be directly applied to enhance the performance of reporter proteins, such as fluorescently labeled proteins, in microbe–drug interaction detection, offering more sensitive tool support for the experimental validation of association prediction results. In microbial interaction network prediction, AI can integrate MAGs data with multi‐omics information to construct network models [175, 176], further providing a theoretical basis and practical framework for microbial community modulation and clinical health intervention design strategies. Although AI substantially accelerates microbial function discovery, predictive performance remains dependent on database completeness and annotation quality. As a result, computationally inferred interaction networks should be interpreted cautiously and complemented by biological validation.

### Artificial intelligence drives the development of biological agents such as antimicrobial peptides

AMPs have traditionally been studied in the context of anti‐infective therapeutics; however, accumulating evidence indicates they may also be considered food‐derived bioactive molecules in dietary settings [177]. Notably, many AMPs can be generated through the enzymatic hydrolysis of dietary proteins, including those derived from milk, eggs, and plant‐based sources [177, 178]. This positions AMPs as naturally occurring components of food‐derived bioactive peptides rather than solely as pharmacological agents. Furthermore, AMPs have been extensively investigated as functional food ingredients and natural preservatives [179, 180], thereby establishing a direct connection between AMPs, food systems, and dietary exposure. Therefore, in the context of this review, AMPs are discussed not as conventional therapeutic drugs, but rather as diet‐related functional biomolecules with potential roles in microbiota regulation and host health modulation.

Conventional antibiotic discovery approaches fail to keep pace with the rapid evolution of drug resistance mechanisms in pathogenic microbes. In contrast, AMPs exhibit selective antimicrobial activity and induce the development of drug resistance at a much slower rate, making them promising candidates as alternatives to conventional chemical antimicrobial agents. However, the rational design of effective AMPs remains a formidable challenge, attributed to the enormous combinatorial sequence space and the imperative to balance therapeutic efficacy with low cytotoxicity. AI enables more efficient exploration of the vast sequence landscape to identify peptides with optimal therapeutic profiles. AI‐driven AMPs design provides a promising avenue for bridging food science and microbiome research. By enabling the rational optimization of peptide sequences and functional properties, AI facilitates the development of targeted bioactive molecules that can be integrated into food systems, either as functional food ingredients or as modulators of gut microbial composition and activity [181]. Currently, two primary application strategies have been established for AI‐based AMP development, namely AMP mining and de novo AMP generation (Figure 3). AMP mining primarily encompasses genomic, proteomic, and MAG datasets, and a series of high‐efficiency AI‐driven screening strategies have been developed for this purpose. State‐of‐the‐art algorithms have successfully identified 2603 potential AMP candidates via systematic scanning of 42,361 protein sequences in the human proteome [182, 183, 184], several of which have been experimentally evaluated and demonstrated therapeutic efficacy in animal models. Beyond this, AI has been extended to mine novel antimicrobial sequences from the proteomes of extinct species and facilitate research in the emerging field of molecular resurrection. For instance, a recent study employed the DL model APEX to mine the accessible proteomes of all extinct organisms, including mammoths, leading to the discovery of multiple novel AMPs such as Neandertalin‐1, Mammothlin‐2, and Elephantin‐2; these peptides have now advanced to preclinical candidate status [185]. In terms of prediction tools, panCleave, a random forest (RF) model designed for genome‐wide proteomic cleavage site prediction, has significantly improved the identification efficiency of candidate peptides [186]. Six publicly available traditional machine learning (ML)‐based AMP models, including Macrel [187], are currently applicable for assisting AMP screening. DL technologies have also emerged as a driving force in advancing this field.

**Figure 3:**
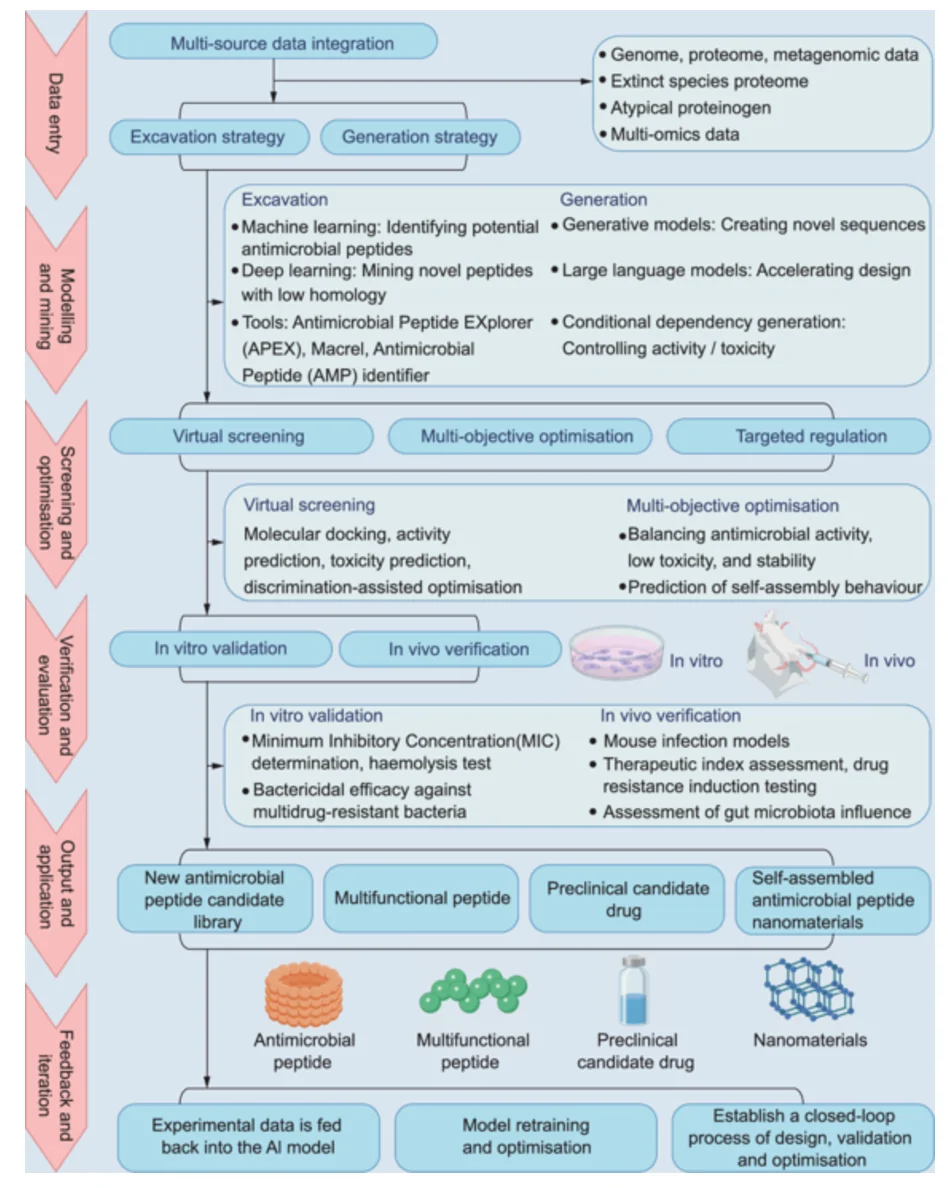
AI‐assisted workflow for the discovery of microbiome‐derived antimicrobial peptides (AMPs). Multi‐omics datasets are integrated to support peptide identification, candidate prioritization, and iterative optimization. AI‐assisted computational prediction is combined with experimental evaluation and validation to refine candidate selection and accelerate the discovery of microbiome‐derived AMPs.

Employing DL techniques such as LSTM and utilizing the human intestinal microbiota as the sequence resource, researchers successfully identified 181 novel AMPs, among which the majority exhibited less than 40% sequence homology with known AMPs and demonstrated significant therapeutic efficacy against drug‐resistant bacterial infections [28]. Beyond sequence‐driven mining strategies, AI has also been integrated into multi‐omics and experimental validation workflows. A milk‐derived AMP prediction model based on light gradient boosting machine (LightGBM), LSTM, and attention mechanism algorithms achieved higher prediction accuracy than existing AMP models in the independent test set [188]; further, using endogenous peptides obtained by LC‐MS/MS analysis via DeepMAMP for prediction, 5 out of 6 peptides exhibited antimicrobial activity. Additionally, AI supports the exploration of peptides from atypical protein sources. Chen et al. [189] adopted an AI‐driven and computationally based high‐throughput approach, combining molecular docking and ML‐based virtual screening, to screen novel dipeptidyl peptidase IV (DPP‐IV) inhibitory peptides from hemp seed proteins. The peptide VAMP not only inhibits DPP‐IV activity but also selectively promotes the growth of intestinal *Akkermansia*, thereby exerting a hypoglycemic effect. More intriguingly, the use of a self‑developed AMP identifier model enabled the screening of cAMPs from the intestinal microbiota of the German cockroach (Blattella germanica), whose antimicrobial activity was subsequently confirmed via both in vitro and in vivo assays [190]. It is important to emphasize that generative AI has the potential to become a crucial tool for peptide generation in the future, as it can learn the underlying sequence distribution and constraints [191]. Currently, the majority of studies have focused on the exploration of Variational Autoencoders (VAEs) or Wasserstein Autoencoders, while Generative Adversarial Networks (GANs) have also been widely applied [13]. It is important to recognize that the evaluation metrics adopted across different AMPs discovery studies are inherently heterogeneous and correspond to distinct biological endpoints. For instance, the minimum inhibitory concentration (MIC) primarily reflects antimicrobial efficacy, whereas the half‐maximal inhibitory concentration (IC50) is commonly used to evaluate cytotoxic or anticancer activity. In addition, parameters such as hemolytic activity and therapeutic index (TI) are employed to assess peptide safety, selectivity, and potential therapeutic applicability. Consequently, these metrics are not directly comparable and should be interpreted within their respective biological and experimental contexts.

Notably, the HydrAMP model enables the generation of entirely novel peptide sequences with high activity and low toxicity through parameterized creativity regulation, and some of these candidates have been evaluated to be effective in animal models [50]. AI‐driven peptide design extends from antimicrobials to anticancer candidates, with auxiliary discriminators optimizing activity and safety profiles [192]. The DL algorithms PepGen 1.0/AmPepGen are capable of generating artificial AMPs, and a variety of highly active synthetic peptides have been obtained through bioinformatics optimization, thereby providing a new strategy for multi‐target anti‐infective therapy [193]. The integration of LLMs has further improved the efficiency: AMP‐Designer combines the pre‐trained AMP‐GPT model with reinforcement learning, successfully designing 18 novel broad‐spectrum AMPs in only 11 days, with an in vitro validation success rate of 94.4% [194]. Gao et al. [195] developed DLFea4AMPGen, an integrated framework that first fine‐tunes a pre‐trained MP‐BERT model to construct an oxidative activity prediction model. This prediction model exhibits superior accuracy compared to traditional models such as Support Vector Machines (SVMs) and CNNs. Subsequently, key feature fragment screening and sequence subspace generation are performed to achieve the de novo design of AMPs, with a success rate of 75%. Controlled generation technology is gradually improving the precision of peptide design. Beyond peptide generation alone, recent AI‐assisted protein engineering platforms have begun integrating large‐scale sequence‐activity mapping with pretrained protein language models to accelerate functional protein evolution. ML‐guided protein engineering can rapidly map sequence–function landscapes from experimentally measured variant data, enabling the prediction and optimization of high‐activity protein variants [196]. Conditional generation informs sequence design by embedding target attributes such as activity and toxicity [197]. In the CLASS model [198], the discriminator is trained on the latent space of the Wasserstein Autoencoder (WAE), enabling the generation of peptides with target activity and toxicity profiles. The closed‐loop generate‐screen‐optimize framework markedly increases the targeting accuracy of biologics development. Additionally, a machine learning algorithm for microbial peptide identification, proposed by Santos‐Júnior [80], greatly reduces false positives in AMP recognition. This algorithm was used to predict new, non‐redundant AMPs and to build the AMPSphere resource, providing a basis for further AMP development.

Encouragingly, the development of AMPs via AI has extended to multiple fields, including antibacterial therapy, with its practical potential well evaluated. However, it must be recognized that most reported performance metrics are derived from in vitro experiments and may not directly translate to in vivo efficacy. Peptides generated by HydrAMP exhibited an MIC of ≤1 μM against drug‐resistant bacteria [14], but their therapeutic efficacy in vivo is influenced by additional factors, including molecular stability, pharmacokinetics, immune interactions, and tissue distribution; AMP‐24, designed by a latent diffusion model, had an MIC of 6.25 μM against multidrug‐resistant Acinetobacter baumannii and exerted significant predicted therapeutic potentials in a murine lung infection model [199]; KW13 and A118 engineered by AMP‐Designer showed a low MIC of 4 μg/mL against Pseudomonas aeruginosa, along with low hemolytic activity and high plasma stability [194]. Via a dual‐channel strategy integrating database mining and text generation, Wang et al. [200] successfully identified cryptic AMPs from Swiss‐Prot and UniProt, and could also de novo generate novel AMP sequences. Peptides developed by Mesa et al. [193] possessed concurrent antibacterial, anticancer, and antifungal activities; among them, OrPIM had an IC50 of <6.25 μM against MCF‐7 cancer cells with a high therapeutic index of 52.6. These cytotoxicity‐related metrics reflect distinct biological mechanisms and should not be directly equated with antimicrobial efficacy. Notably, although AI has greatly expanded the scope of AMPs discovery, standardized evaluation frameworks and improved in vivo validation strategies are still required to comprehensively assess their therapeutic potential. In other models, TransSAFP predicted the self‐assembly behavior and antibacterial function of peptides through transfer learning, and screened the highly efficient self‐assembling AMP p45, which displayed potent bactericidal activity against Methicillin‐resistant Staphylococcus aureus (MRSA) and multidrug‐resistant E. coli. This peptide avoids bacterial resistance via physical disruption of bacterial membrane structures and exerts a Salmonella load‐reducing effect comparable to that of ciprofloxacin [201]. The self‐assembly strategy can further address the stability challenge of AMPs, and nanotechnology affords a more stable and efficient avenue for the functional realization of AI‐designed peptides. In practical applications, AI‐designed AMPs are often hampered by in vivo degradation and low delivery efficiency, and nanotechnology exquisitely mitigates this limitation. Wang et al. [202] found that pH‐and enzyme‐dual responsive nanoliposomes markedly enhanced the antitumor efficacy of the novel peptide Dermaseptin‐PP, which not only reduced its hemolytic risk but also improved its accumulation and penetration in the tumor microenvironment (TME). Revdekar et al. [203] engineered self‐regulating nano‐supramolecular chemical architectures, achieving tumor site‐specific efficacy amplification and controllable release via the dynamic assembly of peptides, small molecules, or peptide‐metal conjugates, which further demonstrated the therapeutic advantages of peptide‐nanostructure conjugation. In addition, AMP nanofibers exert bactericidal effects through a dual mechanism of physical entrapment and membrane disruption, effectively eradicating multidrug‐resistant bacteria and repairing mammary gland tissue in a murine mastitis model [204], thus removing key barriers for the in vivo application of AMP biologics. The combination of AI‐designed candidate peptides with intelligent nanostructures will pave a groundbreaking novel design‐to‐therapy pathway for the engineering and precision medicine application of antibacterial and antitumor peptides. AMPs may influence host health not only through their direct antimicrobial effects, but also by modulating the structure and ecological dynamics of gut microbial communities [181]. This positions AMPs within the diet–microbiota–host axis as potential mediators of microbial selection, community stability, and intestinal ecological balance. In AMPs research, despite substantial advances achieved through individual ML or DL models, certain challenges persist that remain refractory to conventional methodologies. The integration of multiple modeling approaches holds promise for more effectively resolving these issues [12, 50, 80, 186, 187, 193, 194, 195, 200, 201, 202, 203, 204] (Table 5). Despite the rapid progress of AI‐assisted AMP discovery and the encouraging antimicrobial activities observed in in vitro screening systems, several translational barriers remain insufficiently addressed. Most current studies primarily evaluate peptide performance using activity‐oriented indicators such as MIC, half maximal IC50, or TI, whereas key determinants of in vivo applicability—including peptide stability, pharmacokinetic behavior, biodistribution, immunogenicity, toxicity, and host‐related biological constraints—are less consistently evaluated across studies [205]. Moreover, the heterogeneity of evaluation metrics and experimental conditions limits direct comparison between AI‐designed candidates and may complicate cross‐study benchmarking. Therefore, future AI‐assisted AMP development may benefit from incorporating multi‐parameter evaluation frameworks that integrate antimicrobial activity with translational indicators to improve the robustness and practical relevance of candidate prioritization [205].

**Table 5:** Potential model‐combination strategies for AMPs research.

Problem	Model combination	Expected outcome	References
Limited cross‐species and cross‐strain generalization	HydrAMP (generative model) + AMP Scanner Vr.2/CAMPR3 (multi‐classifiers) + transfer learning	HydrAMP generates candidate peptides; multiple classifiers (CAMPR3, AMP Scanner) predict antimicrobial activity across species; transfer learning fine‐tunes models to adapt to newly emerged strains	[50, 193]
Difficulty in balancing antimicrobial activity and toxicity	AmPepGen/PepGen (generative models) + ToxinPred/HAPPENN (toxicity prediction) + HeliQuest (physicochemical optimization)	Generative models propose peptide candidates; ToxinPred and HAPPENN estimate toxicity and hemolytic potential; HeliQuest optimizes hydrophobicity‐hydrophilicity balance to reduce toxicity	[193]
Multi‐objective AMP design	AMP‐Designer (LLM‐based generation) + ENNAACT (anticancer activity prediction) + AmphipaSeeK (membrane interaction prediction)	LLMs generate multifunctional AMP candidates; ENNAACT predicts anticancer activity; AmphipaSeeK evaluates membrane‐binding mechanisms to ensure functional compatibility	[193, 194]
Data scarcity and insufficient negative samples	Macrel (MAGs‐based mining) + self‐supervised learning + active learning	Macrel mines potential negative samples from MAGs; BERT or LSTM pretraining enhances representation learning; active learning iteratively selects informative samples to refine negative datasets	[12, 187]
Limited mechanistic interpretability	PanCleave (cleavage site prediction) + Attention mechanisms (XAI) + molecular dynamics (MD) simulations	PanCleave predicts AMPs candidates from precursor proteins; Attention mechanisms visualize key amino acid contributions; MD simulations evaluate peptide‐membrane interaction mechanisms	[12, 186]
Discovery and revival of ancient or rare AMPs	PanCleave (ancient proteome mining) + AMPSphere (global AMPs database) + HydrAMP (analog generation)	PanCleave predicts AMPs from ancient proteomes; AMPSphere provides reference sequences and activity annotations; HydrAMP generates structurally related analogs with enhanced activity	[50, 80, 186]
Poor in vivo stability and susceptibility to degradation	AMP‐Designer/DLFea4AMPGen + TransSAFP	Simultaneous optimization of antimicrobial activity and self‐assembly behavior, yielding AMPs with improved stability and reduced resistance risk	[194, 195, 201]
Complex metagenomic sequences and long‐range dependency modeling	LSTM (sequence mining) + MP‐BERT/AMP‐GPT (Transformer‐based models) + reinforcement learning	Improves identification and de novo design of low‐homology AMPs, accelerating the generation of high‐activity candidates	[12, 194, 195]
High false‐positive rates in large‐scale AMPs generation	AMP‐GPT/ProteoGPT + low‐false‐positive ML classifiers + AMPSphere	Substantially reduces false positives among millions of generated candidates, enabling the construction of high‐confidence AMPs libraries	[80, 200]
Limited in vivo delivery efficiency and safety of AI‐designed peptides	AMP generative models + ML‐assisted nanostructure design	Enhances in vivo stability and targeted accumulation while reducing hemolysis and off‐target toxicity	[202, 203, 204]

### Artificial intelligence empowers synthetic biology and biosynthesis

Multi‐omics integration provides comprehensive descriptive insights into microbiome–host interactions, whereas synthetic biology represents an emerging strategy for translating these insights into conceptually feasible approaches for targeted microbiome engineering and functional regulation. Synthetic biology is an interdisciplinary field that integrates principles from biology and engineering, aiming to achieve desired functions by designing, constructing, and modifying biological systems. In the field of biosynthesis, AI has accelerated discovery, functional gene mining, metabolic network inference, and protein structure prediction, thereby promoting a shift toward high‐throughput, automated, and rationally designable research. The applications of AI in biosynthesis can be broadly categorized into four modules: tools for identifying BGCs in genomes, tools for functional gene mining, tools for protein structure prediction, and tools for biosynthetic pathway prediction. Although these AI‐based approaches were originally developed within broader biological contexts, they nevertheless provide transferable analytical frameworks for modeling and interpreting diet–microbiota–host interactions. At the upstream stage of biosynthetic research, the primary task of genomic BGC identification tools is to identify gene clusters potentially responsible for the synthesis of specific NPs from microbial genomic or MAGs datasets. The introduction of AI has markedly improved both the efficiency and accuracy of BGC identification. Based on the hidden Markov models (HMMs) of signature proteins, antiSMASH [206] is capable of identifying more than 70 types of BGCs associated with the biosynthesis of antibiotics, anticancer drugs, and other bioactive compounds. By aligning identified BGCs with the MIBiG database, antiSMASH can predict biosynthetic pathways and product structure classes, and further analyze cross‐species homologous gene cluster variation via MultiGeneBlast to explore the potential value of microbial secondary metabolites. DL has demonstrated scalability and effectiveness for BGC mining. For example, e‐DeepBGC integrates convolutional neural networks (CNNs) and bidirectional long short‐term memory (BiLSTM) networks to identify and classify BGCs based on protein‐family domain information. Compared with DeepBGC, e‐DeepBGC achieved improved prediction performance and was subsequently applied to 5666 bacterial genomes, identifying 170,685 BGCs across diverse bacterial phyla [207]. Similarly, Geoffrey et al. [208] developed DeepBGC by integrating BiLSTM and pfam2vec for BGC identification; this tool outperforms ClusterFinder and other conventional methods in terms of BGC detection accuracy and the ability to identify novel BGC categories, with a mean AUC of 0.80 for predicting BGC product categories. DeepBGC applies a BiLSTM‐based deep learning framework to identify BGCs from protein‐family domain profiles, demonstrating the potential of deep learning for large‐scale exploration of microbial biosynthetic diversity [208]. In addition, BGC‐Prophet [209], a Transformer‐based language model for BGC prediction and classification, captures the positional dependencies among genes through a Transformer encoder, facilitating phylogenetically broad and pan‐MAG BGC discovery.

Beyond the gene cluster level, functional gene mining tools are more extensively used to identify genes or elements with specific functions. SYMPLEX is the world's first LLM dedicated to synthetic biology element mining and biomanufacturing applications [210]. By integrating domain‐specific LLM training, synthetic biology knowledge alignment, and large‐scale bioinformatics analysis, it can automatically mine functional gene elements and accurately recommend their engineering application potential. Currently, SYMPLEX has been applied to the mining of capping enzymes, leading to the successful discovery of multiple new high‐performance enzyme species. Notably, beyond DNA‐level regulatory elements, the non‐coding regions of mRNA also exhibit complex regulatory functions that extend well beyond conventional understanding; however, their dynamic interaction networks remain difficult to resolve using traditional experimental and computational approaches. AI‐based methods, including RNA language models, can be used to learn the deep semantic representations of RNA sequences and predict their interactions with molecules such as proteins. In the future, these approaches are expected to construct cell environment‐specific RNA interaction maps [211], thereby providing more comprehensive preliminary information for protein structure and function prediction. Meanwhile, ncRNAs have gradually entered the perspective of AI‐driven biosynthesis research [212]. CircRNAs pose challenges for research due to their low abundance and structural characteristics highly similar to linear RNAs [213]; compared with traditional short‐read methods, long‐read sequencing technology can obtain full‐length circRNAs sequences more directly and accurately [214]. At the functional characterization level, combining differential expression analysis, co‐expression networks, and emerging DL models can be used to predict their cell‐specific expression and associations with diseases [215]. Similarly, based on the BERT‐RBP model, inputting RNA sequences and segmenting them through sliding windows enables the prediction of whole‐transcript RBP‐lncRNA interactions [73]. This model can accurately predict the interactions between mRNAs and known RBPs, as well as the RBP interactions of lncRNAs such as XIST. It can also identify the conservation and differences of related interactions across species, providing a new tool for lncRNA research [216]. In the future, long‐read sequencing can be combined with single‐cell or spatial omics technologies to develop AI models capable of integrating multi‐omics data, which, together with protein structure prediction tools, will provide support for a more comprehensive understanding of biosynthetic mechanisms.

Proteins serve as the core catalysts in natural product biosynthesis. At the structural level, AI‐based protein structure prediction models aim to infer the three‐dimensional conformations of proteins from amino acid sequences, thereby providing a structural basis for understanding enzyme function and guiding rational protein engineering. Represented by RoseTTAFold, the 3‐track neural network has been increasingly applied to protein structure and interaction prediction in biosynthesis‐related contexts [217]. Through three parallel tracks (1D sequence, 2D distance map, and 3D coordinates), it realizes information interaction and integration with the help of attention mechanisms, which is conducive to resolving proteins with unknown functions, assisting experimental structure determination, and accelerating the design of protein complexes. It can quickly generate high‐confidence protein models such as human GPCRs, assist in the molecular replacement resolution of X‐ray crystallography and cryo‐electron microscopy (cryo‐EM), directly predict protein–protein complex structures, and provide a key structural basis for biosynthesis research, such as enzyme design and metabolic pathway optimization. Meanwhile, AI is continuously advancing in nucleic acid structure prediction and protein–nucleic acid interaction models, which will have a profound impact on the integrated understanding of multi‐omics in biosynthesis [218].

On the premise of identified BGCs or target product structures, AI can be applied to predict their specific biosynthetic steps or complete pathways, among which several recently developed tools have demonstrated outstanding performance. BioNavi‐NP is a DL‐based tool designed for predicting the biosynthetic pathways of NPs and natural product‐like compounds, which can help resolve unknown synthetic pathways and reconstruct heterologous synthetic pathways [219]. BioNavi‐NP constructs a synthesis prediction model by integrating a Transformer architecture with biochemical biosynthetic reaction training data, and subsequently enables the efficient generation of plausible biosynthetic pathways through multi‐step pathway sampling using the AND‐OR tree search algorithm (Retro*). The pathway identification rate for test compounds reaches 90.2%, and it can also evaluate pathway feasibility by combining tools such as selenzyme, and provide interactive visualization results through a web‐based interface. Seq. 2PKS [220], on the other hand, first identifies polyketide domains and enzymes through genomic mining, predicts substrate specificity, then takes docking domain sequences as input, combines Probabilistic Neural Network (PNN) search to predict assembly pathways, generates a variety of mature polyketide structures, and screens them through mass spectrometry data. It can accurately predict polyketide structures, discover biosynthetic gene clusters such as monensin, and identify new variants such as 2‐aminobenzamide‐actinophenone, showing excellent performance in the discovery and structure prediction of polyketide compounds. These methods provide new computational solutions for natural product function mining, synthetic biology design, and metabolic engineering optimization. The preceding sections have outlined a range of AI applications in biological systems, many of which are capable of capturing complex, multiscale interactions. Collectively, these approaches provide essential analytical tools for linking dietary inputs to microbial transformations and downstream host physiological responses.

## 4 ARTIFICIAL INTELLIGENCE DRIVES THE TRANSFORMATION OF PRECISION HEALTH PRACTICES

### Dietary regulation modulates intestinal microbiota structure and function

Against the backdrop of the continuous advancement of precision health concepts, dietary intervention design has emerged as a key strategy for improving host health and modulating the intestinal microbiota, owing to its high modifiability and relatively low exploration cost. A substantial body of evidence has demonstrated that diet not only influences the composition and metabolic functions of the intestinal microbiota but also further affects host immunity, metabolism, and disease development processes [221, 222, 223]. However, due to the complexity of dietary components, substantial inter‐individual variability, and the highly dynamic nature of microbial communities, traditional experimental approaches often struggle to accurately resolve the intricate relationships between diet and the intestinal microbiota. Food systems are inherently heterogeneous due to differences in cultivar, geographical origin, processing conditions, compositional profiles, microbial dynamics, and batch effects. Such heterogeneity may increase intra‐class variability, blur inter‐class boundaries, and reduce model generalizability [224]. In this context, AI offers clear advantages in handling such complexity, with different algorithms contributing distinct strengths depending on the characteristics of the data. To address challenges associated with limited sample size and cross‐domain transfer, approaches including data augmentation, federated learning, incremental learning, and transfer learning have gradually been introduced into food research to improve model robustness and adaptability [225]. Moreover, food analysis frequently involves non‐structured data such as spectral and hyperspectral images, where conventional workflows often rely on manual preprocessing [226]. Recent DL approaches have reduced this dependence by enabling CNN to directly extract informative features from raw spectral data [227], while Transformer models further improve the modeling of global spectral relationships and multimodal information in complex food systems [228]. In addition, explainable AI (XAI) methods such as Shapley additive explanations (SHAP) and Grad‐CAM are increasingly adopted to enhance model interpretability and application reliability [229]. Associations between intestinal microbiota and dietary patterns inferred by ML models can facilitate the development of standardized dietary intervention designs targeting the intestinal microbiota and provide more robust scientific guidance for disease prevention. The RF algorithm can evaluate the correlation between different dietary components and intestinal microbiota composition; participants consuming a diet rich in plant‐based foods and high in fiber had a more diverse intestinal microbiota and higher abundances of bacteria producing beneficial SCFAs [15], with longitudinal data confirming strong personalized responses to prebiotics like inulin [230]. CNN can automatically extract intestinal microbiota community structure patterns, with convolutional layers suitable for processing grid‐like data such as dietary images; RNNs have greater advantages in time‐series modeling, enabling tracking of dynamic remodeling processes of the microbiota under long‐term changes in dietary habits; Post‐Hoc interpretable methods such as SHAP are used to reveal the impact of specific features on dietary predictions and address issues such as poor model interpretability [229]. Boodaghidizaji et al. [231] employed four machine learning models (RF, SVM, ANN, and CNN) and found that intestinal microbiota data from fecal samples treated with dietary fiber not only improved the classification accuracy of various diseases but also optimized the differentiation between ulcerative colitis (UC) and CD, demonstrating that ML models can assist in the diagnosis of chronic inflammation‐related diseases through diet. Wang et al. [232] used ML to identify seven key intestinal microbes associated with the multi‐stage modified paleolithic‐ketogenic diet (MDP‐i‐KD), confirming that this diet can regulate the intestinal microbiota. Liu et al. [233] established a linear discriminant analysis (LDA) model based on differentially abundant intestinal microbiota genera, which can be used for disease diagnosis, specifically diagnosing intestinal aging diseases related to high salt (HS). Intestinal aging diseases refer to various diseases caused or worsened by age‐related degenerative changes in intestinal structure and function. Beyond diagnosis, ML models play a crucial role in predictive analysis and biomarker identification. Wang et al. [232] adopted various ML methods and identified *Parabacteroides distasonis* as an intestinal microbiota species associated with weight loss induced by multi‐stage dietary regimens such as intensive ketogenic diet intervention design. Dahlin et al. [234] used ML to determine that Bifidobacterium is the main intestinal microbiota taxon that changes during ketogenic diet (KD) treatment in children with drug‐resistant epilepsy. In addition, Wang et al. [235] used ML models such as SVM and gradient boosting models to identify key intestinal microbiota genera associated with weight loss induced by a low‐calorie balanced diet, including *Blautia*, *Lachnoclostridium*, *Terribacillus*, *Ruminococcus*, and *Pseudomonas*. The gradual introduction of models is promoting dietary prediction research from structural analysis to mechanistic explanation, providing a basic framework for achieving reliable and interpretable prediction of individualized dietary intervention designs. Future strategies may involve a hierarchical approach, utilizing RF for the initial screening of key microbial subsets, followed by refinement using gradient boosting or SVM to construct high‐accuracy models for predicting intervention design outcomes.

### Precision nutritional intervention design improves chronic disease management, aging progression, and mental health

Personalized nutrition is undergoing an AI‐driven paradigm revolution. Traditional one‐size‐fits‐all dietary recommendations have been transformed into a dynamic and predictable precision health intervention design strategy [236]. AI may support hypothesis‐informed and progressively evaluated nutrition strategies. It should be noted that such predictions are based on statistical associations and do not establish causality, requiring further experimental validation. This innovative approach exhibits revolutionary potential in the fields of chronic disease management, anti‐aging and mental health, thereby providing an entirely new avenue for precision health intervention designs. Integrative AI pipelines such as scDock have further extended microbiome‐related therapeutic discovery by combining single‐cell RNA sequencing analysis, intercellular communication inference, and molecular docking, thereby enabling the identification of disease‐associated ligand‐receptor interactions and candidate therapeutic compounds [237]. As an AI‐based dynamic simulation system, the Digital Gut Twin (DGT) [238] has further advanced the development of personalized nutrition. By integrating multi‐layered biological data encompassing microbiome sequencing results, dietary records, host omics profiles and clinical parameters, DGT constructs a virtual replica of the individualized intestinal ecosystem, thus establishing an interactive simulation environment. This cutting‐edge system is shifting personalized nutrition from static dietary advice to a predictable and modifiable dynamic closed‐loop system, and provides a powerful simulation tool for both basic research and clinical practice (Figure 4). The core mechanism of DGT lies in leveraging AI algorithms to decipher the complex interaction network among diet, host and microbiome. This process facilitates the identification of key conceptually feasible intervention design targets, such as specific microbial consortia, functional metabolites and optimal nutritional patterns. At present, most DGT frameworks remain exploratory and require prospective validation. In applied settings, the Q‐net microbiome digital twin model, constructed using longitudinal infant datasets, is capable not only of predicting microbial temporal dynamics and neurodevelopmental risk, but also of simulating potential outcomes under different intervention design strategies [239]. Similarly, AI‐driven platforms such as Gi‐MAPS integrate quantitative microbiome profiling with ecological modeling to simulate microbial community responses under varying dietary or probiotic intervention designs, achieving a high degree of concordance between predicted and observed outcomes [240]. Real‐world deployment and feedback‐driven optimization remain under active development. In clinical applications, multi‐scale digital twin frameworks further integrate microbial dynamics with host pharmacokinetics and have been applied to optimize antibiotic dosing strategies in neonates, highlighting the potential of microbiome‐aware digital twins in precision therapeutics [241]. In addition, related studies have also demonstrated that multi‐scale digital twin models incorporating both microbial dynamics and host pharmacokinetics can be applied to optimize antibiotic therapies in clinical populations [242].

**Figure 4:**
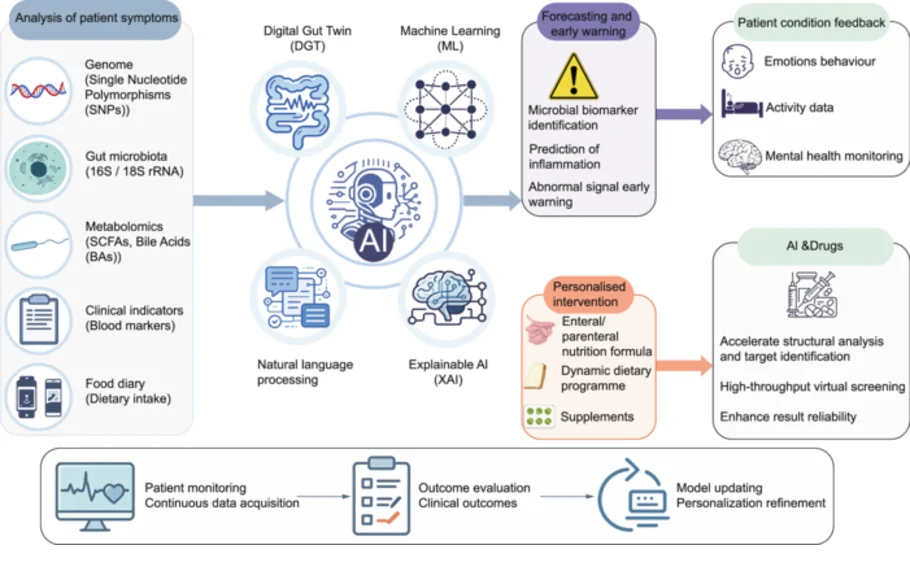
AI‐enabled integration of multimodal patient data for biomarker identification and personalized health management. Multiple sources of patient‐derived data, including genomic information, intestinal microbiota profiles, metabolomic signatures, clinical indicators, and dietary records, are integrated into AI‐based modeling and simulation pipelines. Multi‐omics integration, longitudinal data fusion, ML, natural language processing (NLP), explainable AI (XAI), and mechanistic analysis support hypothesis generation and the identification of clinically relevant biomarkers and biological patterns. The resulting analytical outputs can be applied to disease prediction, biomarker‐based risk stratification, abnormal‐signal detection, and early‐warning systems, thereby enabling timely intervention. AI also supports personalized health‐management strategies, including tailored nutritional and therapeutic recommendations, and may accelerate drug discovery through target identification and candidate screening. The framework incorporates a closed‐loop process of patient monitoring, outcome evaluation, and continuous model updating to improve predictive performance and enable adaptive health management.

In chronic disease management, the role of AI is exemplified by its capacity for the prospective optimization of intervention design strategies. The intestinal microbiota serves as a core target for personalized nutritional intervention designs in chronic diseases; due to the ease of sampling and its distinct diagnostic profiles, microbial components such as bacteria can act as biomarkers for the prediction and diagnosis of various diseases. Studies have demonstrated that the community structure of intestinal fructophilic lactic acid bacteria (FLAB) is significantly associated with colorectal cancer (CRC), cardiovascular diseases (CVDs), and other conditions (AUC = 0.809) [16]. Among these, bacteria such as Streptococcus thermophilus are enriched in healthy populations, indicating their potential as biomarkers for health status or disease risk [189, 243]. In IBD, intestinal microbiota dysbiosis exacerbates intestinal inflammation, while deficiencies in nutrients like vitamin D, zinc, and folic acid disrupt the mucosal barrier and accelerate disease progression [244]. In this landscape, the DGT exhibits extensive value in both research and clinical applications [238], further enabling the transition from diagnosis to intervention design. Specifically, it can be used to predict and simulate the potential impacts of dietary intervention designs on patients' microbiota composition, SCFAs production, immune regulation, and even tumor metabolism. Additionally, DGT supports personalized treatment: in patients receiving anti‐PD‐1 therapy, increasing the intake of fiber‐rich and polyphenol‐rich foods can elevate *Akkermansia* abundance, thereby enhancing treatment efficacy. This development signifies a paradigm shift within nutritional intervention design, transitioning from a traditionally reactive, remedial approach to a proactive, predictive, and regulative framework powered by AI. Furthermore, DGT facilitates adaptive nutrition by continuously updating dietary recommendations through real‐time integration of dietary records, in response to changes in health status, tumor burden, or treatment side effects. Complementing these systems, this approach offers broad utility in ML investigating the interactions among diet, intestinal microbiota, and diseases. Notably, ML algorithms have also been employed to construct diagnostic models for CD; results indicate that models based on intestinal microbiota genes exhibit superior predictive performance compared to those based on species and single‐nucleotide variants [245]. Additionally, studies have explored the association between the intestinal microbiota and CVDs, where five supervised ML models were trained using intestinal microbiota data for the diagnostic screening of CVDs [246]. Similarly, ML methods have been applied to the study of Keshan disease (KD) to clarify the distribution of the intestinal microbiota in affected patients and to understand changes in microbial populations at different disease stages [247], highlighting the potential of ML in delineating microbiota alterations associated with disease progression. Furthermore, studies have demonstrated the crucial role of ML algorithms in elucidating the involvement of the intestinal microbiota in rheumatoid arthritis (RA); findings reveal correlations between RA and taxa, including *Ruminococcus*, *Prevotella*, as well as the species *Ruminococcus bromii* [248]. In addition, ML models can be utilized to explore the association between intestinal microbiota composition in AD patients and AD biomarkers, with results showing that a lower abundance of SCFAs‐producing intestinal microbiota is associated with a higher probability of positive AD biomarkers [249]. Furthermore, AI has promoted the comprehensive precision of nutritional assessment and management. Devices utilizing AI can assist in dietary assessment with high accuracy, and rapid assessment of inpatients can be achieved using acoustic variables, jaw movements, and visual images of chewing and eating [250]. In various diseases, including intestinal failure and cancer, sarcopenia is widely recognized as an important indicator for measuring nutritional status [251]. The application of AI in analyzing muscle imaging enables the efficient identification and quantification of sarcopenic changes. In chronic disease management, AI not only demonstrates excellent ability in biomarker identification but also realizes the optimization of intervention design protocols through DGT, significantly improving the precision and prospectiveness of intervention designs. Meanwhile, it integrates multi‐dimensional health data, can automatically alert abnormalities and optimize management processes, thereby significantly enhancing the efficiency and depth of chronic disease management. Building on this foundation, the application of AI in precision nutrition has gradually expanded from nutritional status assessment to individualized dietary response prediction and dynamic intervention design, thereby providing more refined strategies for metabolic disease management. Studies have shown that postprandial glycemic responses to the same food can vary substantially across individuals, and AI models are capable of accurately predicting individual responses to specific foods. In a randomized controlled trial (RCT), personalized dietary plans designed based on AI predictions led to significantly reduced blood glucose levels [252]. Furthermore, personalized diets derived from machine learning predictions have been shown to be more effective than standard dietary recommendations in lowering blood glucose levels in patients with newly diagnosed T2DM [253]. Beyond glycemic regulation, AI‐assisted personalized dietary intervention designs have also been associated with improvements in multiple cardiometabolic markers, including high‐density lipoprotein cholesterol, triglycerides, and glycated hemoglobin, with intestinal microbiota alterations potentially playing a mediating role in these effects [254].

AI applications extend beyond personalized nutrition into the fields of anti‐aging and mental health, exploring the mechanisms by which nutrition affects the systemic aging process and gut–brain axis function through regulating the intestinal microbiota. In the field of anti‐aging, metabolomics combined with AI clustering analysis technology can identify nutrition‐related characteristics associated with aging. Personalized nutrition provides precise targets for delaying aging by identifying aging‐related biomarkers. The secondary metabolites of FLAB contain antimicrobial/anticancer peptide substances such as RIPP‐like, which have potential anti‐aging effects [255, 256]. Furthermore, integrating metabolomics with AI enables the recognition of nutrition‐related aging characteristics; through clustering analysis, it can be found that low muscle mass is associated with a nutritional pattern of low protein/energy intake and a high carbohydrate ratio, providing directions for intervention design [257]. This method makes up for the limitation of previous studies that only focused on microbiota structure while ignoring functional molecules, and locates the targets of anti‐aging nutritional intervention design from microbiota species to the active substances therein. The metabolic characteristics of the intestinal microbiota are also closely related to the aging process. Studies have found that cognitive decline in the elderly is associated with intestinal microbiota imbalance; the increased abundance of viruses in the intestines, decreased bacterial diversity, and reduced butyrate‐producing bacteria such as *Roseburia* and *Lachnospiraceae* may indicate cognitive decline. Personalized nutritional intervention designs such as the Mediterranean ketogenic diet can regulate the microbiome and improve gut–brain axis function [256]. In addition, non‐coding RNAs (ncRNAs) are gradually becoming quantifiable potential biomarkers for nutrition and diseases; some circRNAs not only exhibit stable expression [258] but also can encode functional peptides and participate in tumorigenesis and metabolic regulation [212, 259], providing new entry points for precise nutrition and intervention design. AI‐driven personalized nutrition programs such as the PROTEIN application can increase microbial richness and diversity by optimizing the intestinal microbiota, thereby indirectly supporting metabolic health [260]. Both Chaudhari et al. [256] and Rouskas et al. [260] took the microbiome as a central element and verified the regulatory role of the microbiome‐mediated AI in aging health through multi‐data analysis. Among them, the whole‐genome MAGs sequencing adopted by Chaudhari et al. provides higher resolution for microbial classification and functional analysis, while the 16S rRNA sequencing used by Rouskas et al. [260] has more advantages in operability and cost control. The two methods jointly improve the technical path of the microbiome in anti‐aging research. On the basis of the above studies, Kloosterman et al. [255] further expanded the application scenario of AI to the excavation of anti‐aging active substances. They developed ML tools based on neural networks, such as SVM, RiPPMiner, and DeepRiPP, to solve the bottleneck of the lack of universal genetic markers in RiPP genomic mining, realizing efficient identification of RiPP precursor peptides and making up for the limitation of previous studies that only focused on microbiota structure while ignoring functional molecules. If combined with the ability of AI to learn the expression patterns of ncRNAs, it is expected to incorporate lncRNAs [261] and circRNAs [262] into individual health prediction models, which, together with the microbiome and metabolome, will form a high‐resolution evaluation system for precise nutrition and disease risk.

In the field of mental health, AI technology has provided unprecedented dynamic analytical tools for nutritional psychiatry, enabling more refined investigation. NLP, by analyzing social media data, can identify the association between emotional eating behaviors and mental health, enabling real‐time population insights [51]. Studies have shown that nutritional biomarkers can predict diseases such as depression. Intestinal microbiota metabolites such as SCFAs can further correlate with mental health by affecting neural function, indicating that personalized nutrition‐mediated microbiota regulation may become a new strategy for mental health intervention design [263, 264]. Population data on the Mediterranean‑DASH Diet Intervention for Neurodegenerative Delay (MIND) diet further support that microbiota‐mediated metabolic changes link dietary patterns to cognitive performance in older adults [265]. ML technology can evaluate the existing multi‐chamber bags component database and quickly determine the most appropriate parenteral nutrition support plan according to the needs of individual patients, taking into account other parameters such as the individual's nutritional status and blood tests. In addition, AI has been proven to be effective in the development and discovery of new therapeutic targets in the pharmaceutical industry [266]. AI modeling algorithms can predict the interactions between drugs and biological targets. Building on such technological advances, personalized nutrition has developed into a thriving research field dedicated to customizing dietary guidance to match each individual's unique biological characteristics. This nutritional approach considers factors such as genetics, metabolomics, intestinal microbiota, lifestyle, and environment, enabling more accurate and effective dietary advice. By applying ML technology, AI algorithms can identify the relationships between individual metabolites, genetic characteristics, and their dietary needs [267]. Meanwhile, AI is also involved in clinical practice. Biofilms are a difficult problem that traditional antibiotics cannot solve; multi‐scale micro‐robots, relying on four driving modes (catalysis, biology, light, and magnetism), can move actively and target biofilms precisely, disrupting biofilm structures, locally releasing reactive oxygen species or antibiotics to achieve efficient sterilization. Such robots have shown excellent potential in treating biofilm infections of devices such as dental implants, catheters, and cardiac stents, providing a new solution for infection management [268].

In the fields of anti‐aging and mental health, AI has revealed the potential mechanisms by which nutrition regulates the intestinal microbiota, influences the gut‐brain axis, and modulates the systemic aging process, expanding the scope of personalized nutrition from physical health to cognitive and emotional management. However, most current ML models are built on cross‐sectional data, excelling at revealing associations rather than causal relationships, and their black‐box nature diminishes the interpretability and credibility of clinical decision‐making. Additionally, the path to clinical translation remains ambiguous, with a significant gap between algorithmic predictions and conceptually feasible, sustainable dietary behavioral changes in individuals, involving multiple links such as user compliance, behavioral psychology, and feasible product delivery forms. Future research on AI‐driven personalized nutrition should focus on two key aspects: first, developing more transparent and interpretable AI models to enhance their clinical utility and physicians' trust; second, conducting large‐scale, long‐term prospective intervention design trials with rigorous designs to verify their long‐term health benefits, cost‐effectiveness, and real impact on public health. To address the issue of insufficient interpretability, when processing metabolite data, Saliency‐Bench can be used to generate saliency maps, visualizing the key metabolites that contribute most to model predictions [269]. Originating from diverse application scenarios, Saliency‐Bench offers a comprehensive framework from dataset construction, multiple types of saliency methods, standardized evaluation to final benchmark test results, providing a solid foundation for measuring the faithfulness and alignment of XAI methods. By promoting the complementary integration of DGT with various ML models and addressing the challenges of interpretability and clinical translation, AI‐driven personalized nutrition is expected to not only revolutionize traditional health management models but also provide strong support for humans to tackle complex diseases and promote healthy aging.

Although digital twin‐related frameworks such as Q‐net, DGT, and Gi‐MAPS demonstrate strong potential for integrating multi‐omics and temporal information, most currently remain at the stage of retrospective analysis, offline simulation, or decision‐support modeling rather than fully deployed closed‐loop systems. Their practical implementation in clinical or agricultural settings still faces challenges, including longitudinal data acquisition, standardized data streams, model updating frequency, and external validation across populations. In particular, real‐time intervention adjustment and automated feedback optimization remain largely conceptual in most existing studies. Future development may require integration with continuous monitoring systems, iterative model recalibration, and prospective validation pipelines to enable adaptive and clinically actionable digital twin workflows. Multi‐model combinations are expected to make up for the shortcomings of single technologies. The development of XAI tools increases the transparency of black‐box models, generating visual maps to highlight the key metabolites with the greatest contribution to enhance clinicians' trust; alternatively, behavioral patterns identified by NLP can be cross‐evaluated with biomarkers discovered by metabolomics to achieve cross‐dimensional integration from psychological to physiological mechanisms [269]. In addition, a monitoring layer is constructed through wearable devices, microbiome and metabolite data, a simulation layer is built with DGT technology to preview intervention design plans, an intervention design layer is created based on algorithms to generate personalized nutritional suggestions, and a feedback layer is established with NLP technology to complete emotional analysis. Through the collaborative application of the above four‐layer technical system, adaptive and sustainable nutritional intervention design is realized.

### Artificial intelligence advances biopharmaceuticals and drug discovery

AI technology is driving revolutionary breakthroughs in the research and development (R&D) of anti‐infective drugs, in a broader conceptual sense, and pharmacologically active drugs can be regarded as a class of nutritional bioactive molecules. Faced with the increasingly severe challenge of antimicrobial resistance in pathogens, AI can efficiently predict pathogen evolutionary trends, identify potential bioactive molecule targets, and accelerate the design and optimization of compounds, which is expected to significantly shorten the conventional R&D timeline from several years to mere months [270]. In recent years, structural biology approaches and high‐resolution structural data have been incorporated into the synthesis of small‐molecule drugs, biomacromolecular drugs, and other bioactive molecules. AI streamlines and optimizes the original R&D workflow by boosting the throughput of structural data generation, cutting the costs of manual molecular modeling, and enhancing the predictive power of computational models. Notably, many of these bioactive molecules can be obtained from dietary sources, including fruits, vegetables, and whole grains. Chen et al. [271] found that in esophageal squamous cell carcinoma (ESCC), the focal adhesion kinase (FAK)/Src family kinase (SFK) axis mediates the phosphorylation of specific tyrosine sites on metabolic enzymes ACLY and ALDOA, driving distinct transcriptional programs that promote primary tumor proliferation and lymph node metastasis, respectively. Leveraging virtual screening approaches, the authors also identified quercetagetin, a naturally occurring dietary flavonoid distributed across multiple plant species. This agent specifically blocks ALDOA tyrosine phosphorylation and potently inhibits the malignant progression of ESCC in both cellular and animal models, thus representing a valuable lead compound and a new dietary‐based therapeutic strategy for managing ESCC metastasis. Yan et al. [272] integrated cryo‐EM with AI‐assisted structure prediction to resolve membrane protein complexes such as Scap‐Insig‐2, constructing more reliable atomic models at medium‐to‐low resolution densities, thereby providing high‐quality inputs for subsequent functional analysis and small‐molecule binding site determination. In addition, their structural analysis of bioactive molecule‐target complexes, such as Nav1.7 and Cav3.2, directly provided a structural basis for small‐molecule design and virtual screening, alleviating the structural bottleneck in drugs and other bioactive molecules discovery and allowing more budget and time to be allocated to candidate molecules [273]. Furthermore, AI can improve process efficiency; emerging methodologies such as CryoSeek integrate automated image screening, clustering, and automatic modeling, facilitating the identification of biological entities and potential targets in larger‐scale and higher‐throughput samples, making the originally time‐consuming process from sample screening to target confirmation more scalable and reproducible, and accelerating the entry of rare or low‐abundance targets into the medicinal chemistry optimization pipeline [274]. Similarly, Su et al. employed high‐resolution cryo‐EM to resolve the conformational underpinnings of large biomolecular machines, adopting an experiment‐centered paradigm in which structures are used to calibrate and evaluate computational predictions. AI provides candidate conformations for experiments, reduces the labor costs associated with isomer classification and density interpretation, and prioritizes experimental validation efforts on high‐value issues [275]. Other investigations have achieved deep integration of AI, supercomputing, and traditional computational chemistry, molecular dynamics, and ML algorithms to address issues such as virtual screening, drug‐target interaction (DTI) prediction [276], drug‐drug (DDI) prediction [277], TCR‐epitope binding prediction, and generative functional peptide design [278]. The aforementioned models leverage AI to improve search efficiency in chemical space screening, interaction prediction, and generative optimization, or possess high‐confidence target structures and experimental validation capabilities. Integrating DTI/DDI [276, 277] with AI‐refined models [273, 275] enables the process from target structure to candidate molecules. A new pocket or a model revised by AlphaFold can be directly used for high‐throughput virtual screening and ML prioritization, followed by the entry of the top N molecules into experimental validation. Meanwhile, Wei's accumulation in interpretable models and supercomputing practices can reduce the black‐box nature of AI outputs and improve experimental tractability [279]. Although these advances have primarily originated from therapeutic and bioengineering applications, they also possess considerable potential for extension to the diet‐microbiota‐host axis, particularly in the identification of diet‐derived bioactive compounds and the development of microbiota‐targeted intervention design strategies.

Nevertheless, AI‐supported precision nutrition has shown considerable promise, while long‐term intervention design evidence and external validation remain limited. Individual variability in diet‐microbiota responses further highlights the need for adaptive and continuously updated predictive frameworks.

### Artificial intelligence promotes the construction of animal health farming systems

At the application level, the One Health concept further extends the diet‐microbiota‐host interaction framework to interconnected human, animal, and environmental systems, emphasizing the integrated nature of health across multiple biological and ecological domains. Targeting the gut‐mammary axis via nutritional microbiota modulation improves lactation performance and udder health, supporting sustainable livestock production [280]. Conventional breeding models rely on empirical decision‐making and face challenges such as antibiotic overuse, high greenhouse gas emissions, and difficulty in quantifying welfare standards. In contrast, AI offers a systematic and novel solution to enhancing efficiency, reducing antibiotics, mitigating methane emissions, and enhancing animal welfare through a process from data perception and intelligent decision‐making to precise execution (Figure 5).

**Figure 5:**
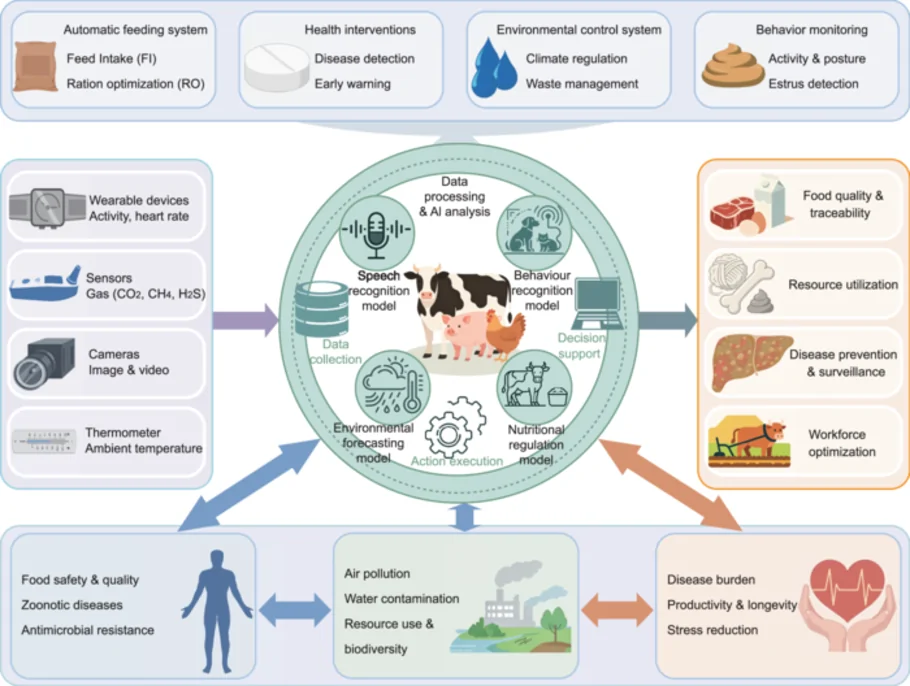
Applications of artificial intelligence in livestock production within a One Health context. This framework illustrates how AI supports livestock production and management from a One Health perspective. Multimodal data collected from wearable devices, environmental and physiological sensors, imaging systems, and temperature‐monitoring platforms are integrated into AI‐driven data processing and analytical workflows. Through behavior recognition, environmental forecasting, nutritional regulation, disease assessment, and decision‐support systems, AI enables automated and precision livestock management. Representative applications include automatic feeding systems for evaluating nutrient requirements and optimizing rations, health‐management interventions for disease detection and outbreak prediction, environmental control for climate and air‐quality regulation, and behavioral monitoring for activity assessment, estrus detection, and stress recognition. These AI‐assisted strategies further contribute to livestock production outputs, including improved food quality and traceability, by‐product utilization, disease modeling and control, and labor optimization. Ultimately, the framework highlights the broader benefits of AI‐enabled livestock management for human health, including food safety, zoonotic disease prevention, and nutritional security; environmental health, including pollution control, resource efficiency, and environmental sustainability; and animal health and welfare, including disease reduction, productivity improvement, and stress mitigation.

The core of efficiency improvement and antibiotic reduction lies in minimizing antibiotic dependence through precision management while concurrently enhancing breeding efficiency. Engineered probiotics such as Bacillus subtilis that protect the intestinal barrier provide an antibiotic alternative, with AI enabling precision delivery at the individual level [281]. First and foremost, pig facial recognition technology achieves individual identification with an accuracy of 96.7% [282]. Combined with the monitoring of dairy cows' rumination behavior via accelerometers, it can dynamically optimize feeding schemes [283], laying a data foundation for precise intervention design. Furthermore, the cough sound recognition model based on DNN‐HMM enables the early diagnosis of porcine respiratory diseases, transforming the antibiotic use pattern from treatment to prevention and significantly reducing therapeutic antibiotic consumption [284]. PLF, through the integration of sensors and AI models, can further promote efficiency improvement and antibiotic reduction. Mathematical modeling methods such as the Hybrid Gray‐Box Model, by integrating mechanistic knowledge with data‐driven approaches, can accurately predict amino acid requirements and feed utilization efficiency, thereby reducing nitrogen and phosphorus emissions while mitigating antibiotic overuse [285].

Methane emissions from ruminants represent one of the major sources of global greenhouse gases (GHGs), posing challenges to sustainable agriculture and climate change mitigation efforts. Technologies such as gene editing, nutritional regulation, and environmental monitoring can all contribute to methane emission mitigation. At the basic research level, recent advances in gene editing technology based on Clustered Regularly Interspaced Short Palindromic Repeats (CRISPR)/CRISPR‐associated proteins (Cas) have enabled direct editing of the methyl‐coenzyme M reductase (MCR) gene in rumen methanogenic archaea, which inhibits the activity of key methane synthesis enzymes [286, 287], providing the possibility of breeding low‐emission livestock breeds at the source. At the practical nutritional level, nutritional regulation also participates in methane control; dynamic nutritional models of PLF reduce GHG emissions caused by nutritional excess by real‐time adjusting dietary components, among which dietary optimization is the key to emission mitigation. AI‐driven dynamic nutritional models, such as the Mills equation, can surpass the limitations of static models such as IPCC Tier 1, accurately optimize rumen fermentation processes by real‐time adjusting the ratio of starch to ADF in the diet, minimize methane intensity (MI) per unit of milk production, and reduce energy loss to as low as 5.93% of the total ingested energy [288, 289]. In addition, AI models can integrate parameters such as forage lipid content; increasing lipid content and secondary metabolites in forage crops can inhibit methane production and improve feed efficiency, with the magnitude of methane mitigation depending on dietary composition and the type and level of lipid supplementation [290], providing intelligent decision support for forage crop breeding and selection. Methane emission mitigation also requires the combination of monitoring technologies to achieve low‐carbonization across the entire breeding chain. Currently, AI intelligent gas sensing systems can filter complex background noise, analyze multi‐component gas spectra through DL models, and perform real‐time edge computing, thereby significantly improving detection stability and sensitivity [291]; a similar concept is also applicable to breeding environment monitoring. The ANFIS model, combined with sensor data, can accurately forecast ammonia and GHG emissions from pig barns and refine ventilation management to reduce carbon emissions [292].

At the level of improving animal welfare, the integration of AI and the Internet of Things (IoT) has constructed an environment for comprehensively perceiving animals' needs and living conditions, transforming welfare assessment from subjective evaluation to objective quantification [293]. It is important to note that while AI models perform exceptionally well in specific agricultural scenarios, the complex breeding environment necessitates multi‐model fusion and collaborative decision‐making. In individual health management, the DNN‐HMM model, specialized in time‐series behavior recognition, can be combined with visual models such as EFMYOLOv3 and YOLOv8n to achieve comprehensive supervision of various animal behaviors, diseases, and growth states; further, inputting the data into the HIMM nutritional model with mechanistic interpretability can realize the transition from early disease warning to precise nutritional intervention design. In environmental and emission regulation, integrating the ANFIS model for environmental monitoring with the Mills equation for emission mitigation, and combining RGB‐D cameras with DL‐based quantitative feed intake technology, enables intelligent regulation that analyzes feeding behavior in conjunction with the environment, and subsequently adjusts dietary formulas to affect methane emissions. Within this framework, cross‐species analyses can help identify conserved microbiome‐associated mechanisms, thereby enhancing the transferability and generalizability of AI models across diverse biological systems.

### Artificial intelligence enhances intelligent monitoring of food safety

Agrifood systems represent an important application domain in which dietary nutrients, microbial ecology, and host health may be coordinately optimized through AI‐driven approaches. The adoption of digital technologies, the deployment of sensors, and the advancement of novel instrumental methods have propelled the development of omics technologies and high‐throughput approaches, which have also found practical applications in food science fields, particularly for process monitoring. The substantial increase in the volume of available data has fostered the development of a wide range of data analysis tools collectively categorized as AI. AI supports risk prediction and decision‐making in food safety management. These tools enable the effective utilization and in‐depth data mining of large‐scale datasets generated by the application of modern technologies, thereby facilitating the establishment of specialized models for food chemical analysis and food safety monitoring (Figure 6A–E).

**Figure 6:**
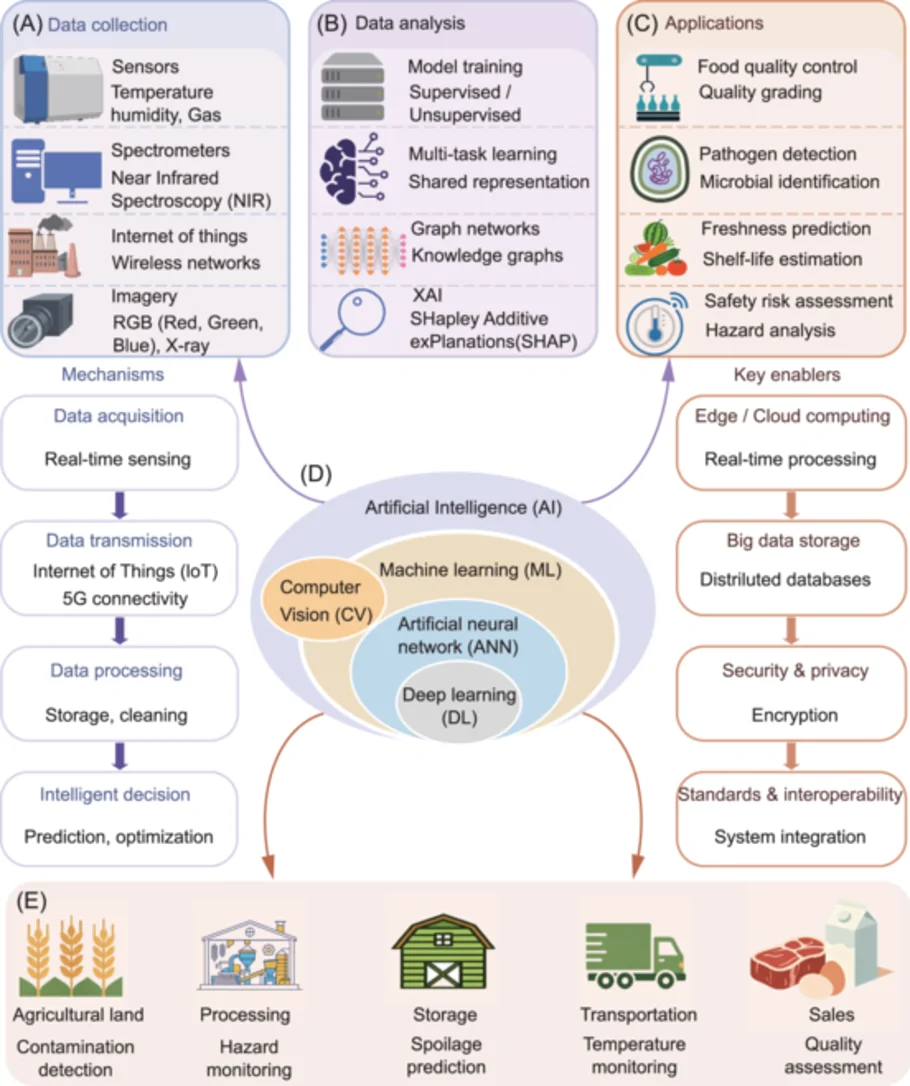
Applications of artificial intelligence in food safety. This framework illustrates the application of AI to food safety management across the full supply chain. The framework encompasses five interconnected layers: (A) data collection, including multimodal acquisition from sensors, spectrometers, Internet of Things (IoT) infrastructures, and imaging systems; (B) AI methods, spanning computer vision (CV), ML, artificial neural networks (ANNs), and DL architectures, with representative approaches including regression, classification, clustering, convolutional neural networks (CNNs), recurrent neural networks (RNNs), long short‑term memory (LSTM) networks, Transformers, graph neural networks (GNNs), transfer learning, multi‐task learning, and XAI; (C) operational mechanisms, involving data acquisition, secure transmission, storage and preprocessing, feature engineering, predictive modeling, intelligent decision‐making, optimization, and feedback control; (D) key enabling technologies, including edge/cloud computing for scalable real‐time processing, big data storage platforms, data security and privacy protection through encryption and access control, and standards for interoperability and system integration; and (E) downstream applications, covering food quality control, pathogen detection, freshness prediction, and safety risk assessment. The framework further illustrates how AI‐driven monitoring supports end‐to‐end management across the food supply chain, from agricultural production and processing to storage, transportation, and sales, enabling contamination detection, hazard monitoring, spoilage prediction, temperature tracking, and final quality evaluation.

In the realm of chemical pollutant monitoring, AI exploration has transformed traditional targeted and single‐item detection into broad‐spectrum and synergistic intelligent risk assessment. In food chemistry, AI technologies facilitate the detailed quantification of chemical constituents, real‐time monitoring of thermal processing curves, and tracking of phase transitions during food manufacturing; these technologies are profoundly revolutionizing food analysis methods, enabling enterprises to ensure the production of higher‐quality and safer food [294]. For pesticide residue detection, conventional methods are time‐consuming and insufficient to address the simultaneous analysis of multiple pesticides. In contrast, the combination of AI‐driven spectral technology and ML models has significantly improved the accuracy and efficiency of detection. SVM have been utilized for the classification and quantitative analysis of several pesticide residues in agricultural products. A study by Liang et al. [10] demonstrated that the SVM algorithm exhibits extremely high accuracy for the classification of pesticide types and the analysis of cypermethrin concentrations. Similarly, Ye et al. [11] constructed a pesticide residue level model using SVM and realized multivariate analysis for the detection of pesticide residue levels in grapes. Other technologies have also been applied in pesticide residue detection: the combination of GANs and hyperspectral imaging technology achieved a residual prediction deviation of 2.7882 for the prediction of pesticide residues in Hami melons, a performance significantly superior to that of conventional models [14]; the combination of one‐dimensional CNN (1D‐CNN) and near‐infrared spectroscopy achieved a 94% recognition accuracy for multiple pesticides on the surface of Hami melons [19, 295]; and the CNN model achieved an even higher detection accuracy of 98.8% for pesticides residues in grapes [18]. In the monitoring of heavy metal contamination, AI can integrate multi‐source data to achieve accurate traceability and quantification. The random forest (RF) algorithm can accurately identify the types and concentrations of heavy metals such as mercury and cadmium in soil by integrating multi‐source data [296]; the DL model WT‐SCAE based on fluorescent hyperspectral technology can simultaneously predict the contents of cadmium and lead in lettuce, with all Rp^2^ values exceeding 0.93 [297]. More importantly, AI provides a new tool for assessing combined pollution risks. In toxicological prediction, quantitative structure‐activity relationship models utilize chemical structure and toxicity databases to rapidly predict the potential toxicity of nanomaterials, additives, and exogenous substances [298], offering an efficient tool for human‐relevant risk assessment. Additionally, it is critical to note that chemical hazard assessment must consider not only the risks posed by individual chemical substances but also the combined risks arising from the interaction of multiple chemical substances. When pesticides and heavy metals coexist in food, they may produce synergistic effects that pose elevated risks to human health [299]. Going forward, models such as multi‐task learning and GNNs could be explored, combining chemical structure and biological effect data to predict the synergistic or antagonistic effects among pollutants and achieve more comprehensive risk assessment.

AI has advanced the frontier of microbial safety prevention and control, shifting from experience‐dependent judgment to data‐driven proactive prediction and real‐time intervention design. It can not only address common threats such as microbial contamination and food spoilage but also perform accurate prediction and real‐time monitoring, thereby bolstering the capacity for early detection and response to potential hazards. This capability further facilitates the optimization of food packaging materials to extend shelf life and reduce food waste [300]. Sharma and Tharani [301] developed a real‐time detection method for foodborne pathogens in fresh agricultural products using optical sensing and ML. Specifically, following the capture of pathogen‐specific optical signals by an optical sensing system, ML algorithms conduct in‐depth analysis and feature extraction on these signals, ultimately achieving rapid and accurate identification of foodborne pathogens. Another important application of AI is multimodal learning, which integrates various data modalities, including spectral analysis, imaging, and omics analysis. By fusing these diverse datasets, multimodal learning can provide a comprehensive and in‐depth understanding of food safety and quality parameters, holding enormous innovative potential [302]. In terms of non‐destructive testing and quality assurance, AI has realized real‐time quality control: CNN can identify low‐concentration foodborne pathogens in fresh food in real time by analyzing optical signals, while the RF algorithm can accurately locate risks by correlating pasteurized milk contamination with factors such as packaging type [20, 303]. AI also plays a role in evaluating food quality through non‐destructive testing, contributing to real‐time quality control and safety assurance by providing accurate and rapid analysis to help manufacturers identify and solve problems [304]. Additionally, AI technologies such as K‐means and DL models can establish prediction models by analyzing historical data combined with environmental variables [305], dynamically assess storage risks, and predict potential food spoilage and contamination risks proactively, which is conducive to preventing foodborne diseases and improving food quality [306].

Food adulteration techniques are becoming increasingly sophisticated and covert, with conventional identification methods often proving insufficient to detect such adulteration. AI‐assisted spectroscopy and sensor technologies can achieve rapid and non‐destructive identification of food adulteration. By integrating cutting‐edge technologies such as spectroscopic analysis, electronic nose (e‐nose), and mass spectrometry, these AI‐assisted approaches provide a revolutionary solution for risk early warning and rapid detection [307, 308]. AI‐assisted Raman spectroscopy has been used for the accurate determination of impurities in food components, emerging as an efficient tool [309]. AI‐assisted mid‐infrared spectroscopy can identify milk adulteration by compositional analysis, thus streamlining quality control in the dairy industry [310]. The combination of near‐infrared and Raman spectroscopy with algorithms such as ML, LDA, Random Forest (RF), and ANNs can accurately identify adulteration cases such as fruit juice adulterated with grape juice, honey adulterated with glucose syrup, and olive oil adulterated with soybean oil [21, 311, 312]. Studies have shown that the combination of Gradient Boosting Classifier and e‐nose can detect adulteration in extra virgin olive oil with an accuracy of 97.75% [313] and can also successfully detect spoilage substances in premium edible oils. Combined with the SVM algorithm, e‐nose can realize non‐invasive real‐time monitoring for the detection of meat adulteration, formaldehyde in milk, and seafood freshness [22, 23]. Additionally, e‐nose has been applied to discriminate coffee flavor profiles and roast intensities, achieving an accuracy of 98% when combined with the ANNs model [24]. AI‐assisted mass spectrometry imaging technology can realize subcellular metabolomic analysis at the cellular level and resolve the complex chemical environment within cells [314]. In food analysis, high‐resolution untargeted mass spectrometry combined with ANNs can distinguish authentic and adulterated foods among wheat varieties and derived products such as bread [315]. AI‐optimized ambient ionization mass spectrometry can rapidly detect trace volatiles in food matrices and improve sensitivity [316].

Collectively, the integration of AI technology with spectroscopy, sensors, and omics data has greatly improved the monitoring capabilities of various links, including pesticide residue identification, heavy metal pollution monitoring, foodborne pathogen early warning, full‐chain traceability, and food adulteration identification. This integration has transformed the originally discrete, lagging detection points into a dynamic, real‐time, and predictive intelligent monitoring framework. However, exploring the complementary integration of multiple AI models can significantly improve the accuracy of overall early warning. For example, the advantages of CNN in feature extraction can be combined with the robustness of SVM for small‐sample classification, or GNNs can be introduced to enhance the ability to analyze complex supply chain relationships. Nowadays, conventional analytical methods continue to serve as the benchmark for confirmatory food safety assessment owing to their established analytical robustness and regulatory acceptance. Rather than replacing these approaches, AI‐enabled analytical workflows increasingly function as complementary tools that improve data interpretation, accelerate screening processes, and extend the practical utility of high‐throughput and real‐time detection platforms [317, 318, 319, 320, 321, 322, 323] (Table 6).

**Table 6:** Comparative characteristics of conventional analytical methods and AI‐enabled analytical workflows in food safety detection.

Evaluation criterion	Conventional analytical methods	AI‐enabled analytical workflows	References
Analytical framework	Quantification and identification based on predefined physicochemical, chromatographic, immunological, or microbiological signals	Computational extraction and integration of high‐dimensional features to support classification, prediction, and decision‐making	[317, 318, 319, 320, 321]
Analytical sensitivity	Generally exhibits high analytical sensitivity and remains the reference standard for confirmatory analysis	Frequently improves practical sensitivity through enhanced signal recognition and extraction of weak or nonlinear patterns	[318, 319, 320, 321, 322]
Analytical specificity	High target specificity under standardized laboratory conditions, although performance may decline in complex matrices	Improved discrimination capability through multidimensional feature integration and model optimization	[317, 319, 321]
Limit of detection (LOD)	Often achieves low detection limits but may require extensive sample preparation and enrichment procedures	Can reduce effective detection thresholds when combined with imaging, spectral, or biosensing platforms	[318, 321, 323]
Detection efficiency	Multi‐step workflows may prolong overall turnaround time	Enables accelerated analysis and supports near real‐time screening and decision‐making	[318, 319, 320]
Economic considerations	High expenditure associated with instrumentation, consumables, and specialized personnel	Higher initial investment in model development, with reduced marginal cost and improved scalability during deployment	[317, 320, 322]
Sample throughput	Usually constrained by laboratory processing capacity	Supports automated and large‐scale analysis with improved throughput	[318, 319, 322]
Operational dependency	Performance is closely linked to operator expertise and protocol standardization	Reduces subjective interpretation and improves inter‐operator consistency	[317, 318, 321]
On‐site applicability	Primarily laboratory‐based with limited portability	More adaptable to portable devices and continuous monitoring systems	[318, 323]

## 5 ARTIFICIAL INTELLIGENCE ADDRESSES CHALLENGES IN DIET‐INTESTINAL MICROBIOTA‐HEALTH RESEARCH

Addressing the application bottlenecks of AI in the diet‐microbiota‐health axis necessitates equal emphasis on technological innovation and ethical governance. Through organ‐level mechanistic validation, digitally optimized intervention design, and global risk management and control, we can realize the shift from precision prediction to responsible intervention design, thus advancing toward a healthier future characterized by the symbiosis between humans and microbiota. The following section synthesizes recurring limitations that have already been introduced within individual application contexts and discusses their shared implications for future translational development.

### Technical limitations constrain the application of artificial intelligence models

The most prominent technical challenges currently confronting AI in relevant research fields converge on the integration of multi‐omics data—including genomic, microbiomic, and metabolomic data—and are manifested in three key aspects: the prevalence of data silos, inadequate mechanistic elucidation, and formidable inter‐species translatability barriers. Multi‐omics datasets are commonly plagued by inherent limitations such as small sample sizes, the absence of longitudinal follow‐up, and marked inter‐cohort heterogeneity, which render numerous AI models difficult to replicate across more diverse populations [125, 126]. Notably, ncRNAs exhibit distinct expression characteristics of strong tissue and organ specificity [258], as well as strict temporal and environmental dependency [324]; their detection is highly reliant on specific sequencing strategies and analytical algorithms, which further exacerbates data heterogeneity in multi‐cohort and multi‐platform studies and thus imposes more stringent requirements on the development and application of AI models. Owing to the heavy reliance of most existing studies on small‐sample, single‐time‐point datasets, it is difficult to capture the dynamic temporal and spatial fluctuations of the microbiota, thereby leading to poor inter‐cohort generalizability and reduced predictive performance of AI models for IBD [125]. Furthermore, although studies based on MAGs can effectively distinguish IBD patients from healthy individuals with an AUC greater than 0.90, sample selection bias inevitably restricts the stability of the identified diagnostic and predictive biomarkers [126]. Similar technical predicaments persist in the broader research domains of food safety and dietary assessment: insufficient sample standardization, a scarcity of negative samples, and interference from extrinsic variables collectively limit the practical applicability of approaches such as generative design and image recognition in real‐world scenarios, with their performance being significantly hampered by factors including image quality and the variable shapes and colors of food products and their packaging containers [325, 326]. To address current limitations associated with data silos, limited sample size, and cross‐cohort heterogeneity, future research may move toward standardized and longitudinal multi‐omics integration frameworks [327, 328]. At the data level, harmonized preprocessing pipelines, unified metadata annotation, and cross‐study quality control procedures may improve reproducibility across cohorts and platforms. At the integration level, metagenomic profiles can provide microbial compositional and functional context, metabolomics can capture downstream biochemical outputs, and ncRNA datasets may contribute host‐level regulatory information. Integrative analytical strategies combining feature alignment, latent representation learning, and network‐based modeling may facilitate cross‐platform data fusion and improve biological interpretability [327]. Furthermore, longitudinal modeling approaches incorporating temporal trajectories may enable dynamic characterization of diet–microbiota–host interactions and reduce the limitations of static cross‐sectional inference [328]. Such frameworks may ultimately support more robust and transferable AI‐assisted microbiome research while still requiring prospective validation across independent cohorts.

Currently widely used microbiome databases, such as the Human Microbiome Project (HMP), have established a reference system covering microbial flora, metabolomic, and immune indicators [329]; however, they contain limited diet‐related information, making it difficult to support research on the associations between diet and the microbiome. Similarly, databases such as MGnify [330] and GMrepo [331] provide standardized intestinal metagenomic datasets but still lack systematic links to individualized dietary behaviors, nutrient intake, and food structure information. As a result, AI models are often constrained to quasi‐static predictions of the microbiome and face difficulties in capturing the dynamic changes in dietary inputs over time. In the field of food science, databases like FDB [332] and USDA FoodData Central maintain comprehensive structured records of food components and nutritional compositions, yet they lack a connection layer with intestinal microbiota phenotypes and host metabolic data, posing significant challenges to cross‐domain integration. The current insufficient mechanistic elucidation is reflected in the obvious black‐box characteristics of most AI models, which hinders the revelation of host‐microbe interaction mechanisms and leaves the causal relationships between the microbiome and host genes unclear. Moreover, the absence of standardized modeling pipelines, consensus hyperparameterization strategies, and transparent model construction process makes it difficult to establish broadly accepted methodological norms. This not only impedes the evaluation by regulatory authorities but also affects users' trust in AI assistance. Furthermore, the lack of high‐quality public datasets in fields such as precision nutrition means that many advanced algorithms can only rely on small‐scale self‐built databases by researchers, which further limits the generalizability of the models [333, 334].

Furthermore, barriers to inter‐species translation impose more in‐depth limitations. In AMPs design, AI‐generated sequences often exhibit ideal performance in animal experiments, prioritizing candidate AMP discovery for downstream functional validation. For instance, AMP‐24 shows favorable MIC values in drug‐resistant Acinetobacter baumannii models, but its translation rate decreases significantly in human clinical trials [335]. Such differences stem from inter‐species variations in the immune microenvironment and insufficient stability of chemically modified peptides in the human body [336]. Future breakthrough directions may involve integrating longitudinal multi‐omics data with XAI to construct dynamic community metabolic models such as CoCo‐GEMs, thereby revealing key nodes of metabolic networks and microbial community dynamics through more interpretable model structures; alternatively, multi‐modal integration and standardization tools such as molecular dynamics simulation and Standard‐GEM can be utilized to further enhance the reliability of models and their applicability across biological systems [337, 338].

### Ethical issues restrict the application boundaries of artificial intelligence

In addition to technical challenges, the ethical controversies triggered by the practical application of AI call for urgent and systematic governance. At present, the core issues can be summarized into three dimensions: the lack of algorithmic fairness, the ambiguous delineation of the ownership of microbial data, and the lag in the construction of regulatory frameworks. First and foremost, imbalances in algorithmic fairness are particularly prominent. Taking the AI‐based personalized nutrition program PROTEIN as an example, although this program can significantly improve intestinal flora diversity [208], its adaptability in resource‐scarce regions is significantly reduced because most of its training data are derived from high‐income populations [339]. Moreover, if the subjective biases of developers are integrated into algorithm design, it will also lead to unfair decisions; for instance, AI may assign African Americans to inconvenient medical appointment times due to biased historical data [340]. Meanwhile, the application of AI in flora research and medical fields is highly dependent on multi‐dimensional patient data, but the actual scope of exploration of data usage and consent statements is still very limited, posing severe challenges to ethics and privacy protection. Second, the ambiguous delineation of microbial data ownership has become another major obstacle to the application of AI in flora research. The generation and utilization of fecal MAGs data involve the legitimate rights and interests of multiple stakeholders, including donors, researchers, and enterprises. Although AI technology has been employed to optimize the donor screening process for FMT [341], existing legal frameworks have not yet clarified whether microbiome data should be classified as personal medical information. This ambiguity not only introduces significant uncertainty into the commercial application of FMT screening and flora‐targeted intervention design technologies but also significantly increases disputes related to patent applications and benefit distribution of relevant technologies. Thirdly, the lack of a regulatory framework has further amplified the ethical risks of AI applications in flora research and food nutrition fields. The field of ML still lacks authoritative industry standards for evaluating algorithm performance and safety. Although food safety AI early warning systems, such as blockchain‐based traceability, can effectively improve the transparency of the food supply chain, the potential abuse of their algorithms may lead to false alarms due to misuse or miscalibration. For example, even though the accuracy of electronic nose technology in identifying meat adulteration can reach 98.1% under controlled conditions [22], it may still cause misjudgments in detection results due to algorithmic biases when operating in actual supply chain scenarios.

In summary, it is imperative to construct a regulatory framework for AI applications centered on transparency, fairness, and meaningful human oversight. Specifically, it is first necessary to establish the principle of “common ownership” of microbial data and incorporate this principle into the unified standards of algorithm auditing. Meanwhile, it is essential to ensure the transparency and compliance of algorithmic decisions through dual technical and institutional means [342], and rely on blockchain technology to realize the full‐process traceability and refined management of the ownership of microbial data [303]. On this basis, to mitigate data and algorithmic biases from the source, it is necessary to carry out standardized data cleaning before AI model training [343], and diverse training datasets should be employed to support regular bias detection and optimization of the model.

### Multimodal technology synergy constructs a systematic research paradigm

Against the backdrop of multiple coexisting challenges, technological integration is emerging as a key direction driving the development of the field. In recent years, cutting‐edge technologies such as organoid technology, digital twin systems, spatial multi‐omics, and multimodal DL have been gradually contributing to the construction of a more robust research paradigm. Microbiota‐gut‐brain axis multi‐organ chips enable in vitro validation of AI‐designed peptides and drug candidates, complementing organoid‐based mechanistic studies [344]. Furthermore, the combination of humanized organoids and AI‐predicted probiotics allows quantitative assessment of the effects of microbial metabolites, such as GABA on neuroinflammation, and also provides a novel experimental platform for the intervention designs of neurodegenerative diseases such as PD [345]. At the same time, microbial digital twin systems integrate genomic and metabolomic data through personalized Co‐GEMs to achieve dynamic simulation of intervention design effects. Notably, the AI framework MICOM can accurately predict SCFAs metabolic functions, providing guidance for synthetic flora design [160], while knowledge graph‐based approaches, such as AEPM, further construct the association networks among peptides, microbes, and diseases, thereby facilitating more precise AMPs design [9]. Furthermore, the analytical capability of spatial multi‐omics is reshaping approaches to investigating microbe‐host interactions [25]. The DL model soScope can significantly enhance the resolution of spatial multi‐omics, and by realizing joint enhancement of spatial multi‐omics data [29], it provides a foundation for driving the iterative optimization of digital twin systems [337, 338] and improving the prediction accuracy of interaction networks [334]. Meanwhile, the integration of AI with advanced sensors and IoT to develop intelligent detection equipment is realizing a real‐time monitoring system for the entire chain of food production, processing, and transportation, transforming food safety from traditional random inspection to continuous and full‐process dynamic surveillance. Beyond concerns regarding fairness, data ownership, and regulatory lag, implementation‐oriented governance mechanisms remain underdeveloped in microbiome and nutrition research [346, 347]. One practical challenge involves ownership and reuse of fecal MAG data. Since fecal‐derived microbial genomes originate from host‐associated biological samples but generate population‐level scientific value after computational reconstruction, an exclusively individual‐ownership model may not be sufficient [348]. A more feasible approach may involve a layered governance framework in which participants retain rights over sample donation and consent management, whereas de‐identified MAG‐derived resources are managed through controlled‐access repositories with transparent data‐use agreements, standardized metadata requirements, and benefit‐sharing principles [346, 347, 348]. Under this model, downstream AI model development and nutritional applications could remain open for research while maintaining accountability, reproducibility, and participant protection [346]. Such governance mechanisms may provide a practical direction for balancing innovation and responsible data stewardship in future microbiome‐based precision nutrition systems [347, 348].

To address these limitations, recent studies have increasingly incorporated causal inference strategies into AI‐based analytical frameworks [349]. Longitudinal datasets, intervention design‐based studies, and perturbation experiments can provide temporal and directional evidence that is often absent in purely correlation‐driven models [350, 351]. At the same time, integrating mechanistic knowledge with machine learning approaches may further enhance model interpretability and facilitate a more rigorous understanding of the underlying biological relationships [352]. Collectively, these developments suggest that future progress in the field will depend not only on advances in algorithmic performance but also on the deeper integration of data‐driven methodologies with hypothesis‐informed biological research.

## 6 CONCLUSIONS AND FUTURE PERSPECTIVES

The integration of diet, AI, intestinal microbiota, and host health should be conceptualized as a closed‐loop regulatory system, in which AI serves as the analytical core that connects nutritional inputs with downstream biological outcomes. This review establishes and elaborates on the novel AI‐driven four‐dimensional integration paradigm of diet‐AI‐intestinal microbiota–host health, highlighting its transformative value in nutritional health research. Intestinal microbiota serves as the core link mediating dietary signals to maintain host homeostasis, exerting regulatory effects through metabolites such as SCFAs and functional components like postbiotics. AI has emerged as a pivotal technical support, breaking through traditional research limitations by integrating multi‐omics data to construct microbial metabolic models, predict interspecific interactions, and drive informed optimization of diet‐microbiota crosstalk. Among these approaches, multi‐omics integration and metabolic network modeling have already demonstrated a relatively high level of application maturity to a certain extent. This paradigm has facilitated the transformation of precision health practices, covering chronic disease management, animal health breeding, and food safety monitoring. However, the role of AI in mechanistic interpretation and practical intervention design remains largely confined to hypothesis generation and candidate prioritization. Translating these predictions into mechanistic understanding and controllable intervention designs still depends heavily on further experimental validation and systematic integrative research. At the same time, the underlying signaling pathways of microbiota‐host interactions remain incompletely elucidated, and AI applications face technical constraints and ethical challenges that require targeted solutions.

Future development should move beyond correlation‐driven modeling toward integrated frameworks that combine predictive AI with causal inference approaches and experimental validation. Potential directions include incorporating longitudinal multi‐omics data, intervention‐derived datasets, counterfactual modeling, and perturbation‐based experimental systems to distinguish causal drivers from correlated signals. Under such a framework, AI would function not as a direct mechanism‐discovery tool, but as a prioritization layer that generates testable hypotheses for downstream validation and application. This transition may provide a more robust foundation for advancing precision nutrition, functional biomolecule design, and food‐related health applications. The four‐dimensional integration paradigm with key priorities as follows should be advanced. First, prioritize mechanistic studies to clarify the core signaling pathways of microbiota‐host crosstalk and the dynamic regulatory mechanisms of the gut *X*‐axis network in integrating diet–microbe–host signals, while carefully considering factors such as the species, age, and physiological state of experimental subjects to avoid confounding effects. Second, optimize AI algorithms adapted to multi‐omics data characteristics and establish standardized data platforms to improve the accuracy and generalization of microbial function prediction, expanding AI applications in biological agent development and synthetic biology innovation. Third, strengthen the formulation of standardization and ethical norms, including unified standards for postbiotic production and functional evaluation, as well as guidelines for data security and privacy protection in AI‐driven precision health research. Fourth, accelerate the industrial transformation of research findings, translating theoretical advances into personalized nutritional products, green animal feed additives, and intelligent food safety monitoring systems. Finally, promote in‐depth interdisciplinary cooperation among microbiology, AI, nutrition, and clinical medicine, and expand international collaboration to establish unified technical standards, thereby advancing the global application of the four‐dimensional paradigm and driving revolutionary development in the nutritional health and biological industries.

## AUTHOR CONTRIBUTIONS

**Tianle He**: Conceptualization; investigation; writing—original draft; writing—review and editing; visualization; validation; methodology; software; formal analysis. **Shuobo Zhang**: Investigation; conceptualization; methodology; visualization; software; data curation; writing—original draft; writing—review and editing. **Jiaxin Chen**: Formal analysis; software; methodology; investigation. **Xiaoling Zheng**: Conceptualization; investigation; formal analysis; software. **Jixin Zhao**: Conceptualization; investigation; methodology; validation. **Huifeng Li**: Investigation; validation; formal analysis; software. **Jiani Mao**: Formal analysis; software; methodology; investigation. **Ju Luo**: Conceptualization; investigation; formal analysis; validation. **Jundan Zheng**: Writing—review and editing; visualization; formal analysis. **Dengjun Ma**: Formal analysis; validation; writing—review and editing. **Lihong Wang**: Validation; writing—review and editing. **Junhui Liu**: Formal analysis; software; visualization; writing—review and editing. **Wen Tian**: Methodology; validation; writing—review and editing. **Shuangming Yang**: Formal analysis; validation; visualization; software. **Chengli Liu**: Formal analysis; software; methodology; writing—review and editing. **Ke Tian**: Formal analysis; software; visualization; validation; writing—review and editing. **Zhenguo Yang**: Conceptualization; investigation; funding acquisition; writing—review and editing; visualization; validation; methodology; formal analysis; project administration; resources; supervision; data curation.

## CONFLICT OF INTEREST STATEMENT

The authors declare no conflicts of interest.

## ETHICS STATEMENT

No animals or humans were involved in this study.

## Data Availability

Data sharing is not applicable to this article as no datasets were generated or analyzed during the current study. No new data and scripts were generated for this review. Supplementary information (graphical abstract, slides, videos, Chinese translated version, and updated materials) may be found in the online DOI or iMeta Science http://www.imeta.science/imetaomics/.
